# Gene Expression Profiling in the Injured Spinal Cord of *Trachemys scripta elegans*: An Amniote with Self-Repair Capabilities

**DOI:** 10.3389/fnmol.2017.00017

**Published:** 2017-02-07

**Authors:** Adrián Valentin-Kahan, Gabriela B. García-Tejedor, Carlos Robello, Omar Trujillo-Cenóz, Raúl E. Russo, Fernando Alvarez-Valin

**Affiliations:** ^1^Department of Cellular and Molecular Neurophysiology, Instituto de Investigaciones Biológicas Clemente EstableMontevideo, Uruguay; ^2^Molecular Biology Unit, Institut Pasteur de MontevideoMontevideo, Uruguay; ^3^Departamento de Bioquímica, Facultad de Medicina, Universidad de la RepublicaMontevideo, Uruguay; ^4^Sección Biomatemática, Unidad de Genómica Evolutiva, Facultad de Ciencias, Universidad de la RepúblicaMontevideo, Uruguay

**Keywords:** spinal cord injuries, genomics, RNAseq analysis, turtles, amniotes, animal models, axon regeneration, glial scarring

## Abstract

Slider turtles are the only known amniotes with self-repair mechanisms of the spinal cord that lead to substantial functional recovery. Their strategic phylogenetic position makes them a relevant model to investigate the peculiar genetic programs that allow anatomical reconnection in some vertebrate groups but are absent in others. Here, we analyze the gene expression profile of the response to spinal cord injury (SCI) in the turtle *Trachemys scripta elegans*. We found that this response comprises more than 1000 genes affecting diverse functions: reaction to ischemic insult, extracellular matrix re-organization, cell proliferation and death, immune response, and inflammation. Genes related to synapses and cholesterol biosynthesis are down-regulated. The analysis of the evolutionary distribution of these genes shows that almost all are present in most vertebrates. Additionally, we failed to find genes that were exclusive of regenerating taxa. The comparison of expression patterns among species shows that the response to SCI in the turtle is more similar to that of mice and non-regenerative *Xenopus* than to *Xenopus* during its regenerative stage. This observation, along with the lack of conserved “regeneration genes” and the current accepted phylogenetic placement of turtles (sister group of crocodilians and birds), indicates that the ability of spinal cord self-repair of turtles does not represent the retention of an ancestral vertebrate character. Instead, our results suggest that turtles developed this capability from a non-regenerative ancestor (i.e., a lineage specific innovation) that was achieved by re-organizing gene expression patterns on an essentially non-regenerative genetic background. Among the genes activated by SCI exclusively in turtles, those related to anoxia tolerance, extracellular matrix remodeling, and axonal regrowth are good candidates to underlie functional recovery.

## Introduction

Spinal cord injury (SCI) is the cause of one of the most devastating conditions for humans because leads to permanent loss of nervous functions below the site of injury. Considerable research efforts have been made to find new therapies aimed to reduce (or revert) damage or to improve the quality of life of patients living with SCI (Silva et al., 2014). Some groups explored the use of mesenchymal (Park et al., 2004; Kanno, 2013; Li and Lepski, 2013) and neural stem cells (Sandner et al., 2012), whereas others focused on gene therapy. The latter approach appears as a promising alternative oriented to stimulate intrinsic repairing mechanisms of the injured cord (Weidner et al., 2012). In this regard, special attention has been paid to the modulation of diverse cell growth factors. Among them, those from the neurotrophin gene family (particularly BDNF that promotes axonal growth) yielded the most promising results in relation to therapeutic possibilities. However, currently available gene therapy treatments do not achieve a significant functional recovery (Neirinckx et al., 2014). Moreover, complications from potential treatments, such as an increased risk of tumorigenesis (Lee et al., 2013), have hampered possible clinical applications.

The inability to regenerate the spinal cord is not restricted to adult mammals, but is also observed in birds, most reptiles, and even in non-amniotes like frogs (Diaz Quiroz and Echeverri, 2013). In non-amniote vertebrates, regeneration of the spinal cord is more common. Among the latter, urodele amphibians (salamanders) appear as one of the most notable examples, as they regenerate several parts of their body after amputation, including the spinal cord (Tanaka and Ferretti, 2009; Diaz Quiroz and Echeverri, 2013). In fact, 2.5–5 months after spinalization, salamanders recover the ability to walk and swim almost as uninjured animals (Chevallier et al., 2004; Cabelguen et al., 2010). Some Osteichthyes (bony fishes) can also regenerate their spinal cords. Such is the case of the model species *Danio rerio* (Zebrafish), yet its regeneration ability is more limited than in salamanders (Becker et al., 1997).

So far, the only known amniotes with spinal cord regenerating capacity are the slider turtles, a group of American freshwater turtles commonly used as pets (Rehermann et al., 2009, 2011). The phylogenetic location of this group implies that they are the closest relative to mammals exhibiting this feature. This evolutionary aspect is an essential consideration that makes these turtles a unique model to investigate the peculiar cellular and molecular mechanisms that allow regeneration or reconnection in some vertebrate groups but not in others.

The cellular and functional processes that take place after traumatic spinal injury in turtles were characterized by Rehermann et al. (2009) in the species *Trachemys dorbignyi*. Briefly, the first histological indications of healing appear about 5 days after injury, consisting in the formation of a cellular bridge permissive for growing axons, which connects the cephalic and caudal portions of the injured cord. Although, full anatomical restoration and functional recovery was never observed, given a proper recovery time, turtles are able to walk again using their four legs though stepping is slower than in normal animals. Moreover, their hindlimbs are not employed for swimming (Rehermann et al., 2009). Therefore, this group of turtles represents an intermediate stage between non-regenerating vertebrates, like mammals, and fully regenerating animals like most anamniote vertebrates. In summary, the crucial phylogenetic location of turtles (relative proximity to mammals) and their incomplete regeneration capacity are strong reasons to consider this group a unique model to study spinal cord regeneration.

Most previous molecular studies on the response to SCI were focused on limited sets of genes, which were known or suspected to have a direct correlation with the process (van Horssen et al., 2006; García et al., 2012; Demircan et al., 2013; Verslegers et al., 2013). However, considering the complexity of SCI response, both in regenerative and non-regenerative vertebrates, it would be also important to tackle its study from a genome wide perspective rather than focusing only on some potential candidate genes.

At present, there is no genome-scale study characterizing the response to SCI in turtles, and to the best of our knowledge, neither there is any global comparative analysis among other vertebrate groups (having different or similar regenerating abilities). Besides, genome scale studies on mammals are scarce with just some assessments in rodents (Velardo et al., 2004; Chen K. et al., 2013; Torres-Espín et al., 2013). In this work, we explore the gene expression patterns (in a genome wide scale) associated with the response to SCI in turtles, aiming to understand the molecular mechanisms that allow regeneration in these animals but are absent or inhibited in mammals.

## Materials and methods

### Animals and surgical procedure

Fresh-water turtles of the species *Trachemys scripta elegans* (carapace lengths 6 cm) were used (*n* = 12) in this study. The detailed surgical procedures and post-operative care have been described previously (Rehermann et al., 2009). As control we used “sham injured” animals that were obtained using the same surgical procedure as in actual injured animals, but leaving the spinal cord intact. This control allowed us to “subtract” the side effects of surgery on gene expression from those caused by SCI itself. All animal handling and experimental procedures were performed according to the animal welfare guidelines of CNEA (National Commission for Animal Experimentation). The protocols were evaluated and approved (license # 005-09-2015) by the CEUA-IIBCE (Comisión de Ética en el Uso de Animales), the Committee for Animal Care and Use of Instituto de Investigaciones Biológicas Clemente Estable.

### Tissue preparation

For RNASeq differential expression analysis we studied a total of 9 turtles that were killed 4 days after surgery. In injured turtles, a 4 mm long cord segment of the spinal cord centered on the lesion epicenter was quickly dissected out and placed in RNAlater (Sigma-Aldrich, St. Louis, MO, USA). Spinal cord segments of sham injured animals were processed in the same way as those with a spinal cord transection.

### RNA extraction, library preparation, and sequencing

The tissue was mechanically homogenized using a cordless motor and pellet pestle (Sigma–Aldrich). Total RNA was extracted and purified using TRIZOL reagent (Invitrogen, Carlsbad, CA, USA) following the manufacturer's protocol. The concentration of RNA samples were quantified using the Nanodrop (Nano-Drop Inc., Rockland, DE, USA) and RNA integrity was determined using Bioanalyzer (Agilent Technologies, Santa Clara, CA, USA). Only samples with a RNA Integrity Number (RIN) higher than 7 were used to make sequencing libraries. RNA samples were polyA selected and sequencing libraries were constructed using ScriptSeq RNA Sample Prep Kit as described in the ScriptSeq RNA Sample Preparation V2 Guide (Illumina). ScriptSeq Index PCR primers were used to allow sequencing several libraries in a single lane. In total, six samples were sequenced, two derived from sham animals, three from injured ones, and one sample which consisted on a pool of different tissues. The latter was used only for transcriptome assembly (see Section *De novo* Transcriptome Assembly). Two replicates, one derived from sham and the second from injured animals consisted in a pool of 3 specimens each and were sequenced using 2 × 100 paired-end reads in an Illumina GAIIX platform. Three additional biological replicates (one sham and two spinalized animals) were derived from one specimen each, and were sequenced using 2 × 75 paired end reads in Illumina MiSeq system. RNASeq data used for this study was deposited in the SRA repository under the accession number SRP082501.

### Gene expression analysis

The genome of a close relative species, *Chrysemys picta*, was combined with *de novo* transcriptome assembly (from *T. scripta* itself) to obtain a mapping reference. The assembled transcriptome allowed us to analyze genes which are too divergent between the two species (hence preventing read mapping on *C. picta*) or not present in the *C. picta* genome assembly.

#### Improving the annotation of *C. picta* (reference) genome using RNAseq data

Reads were mapped to the *C. picta* genome (NCBI cpic v 3.0.1) with Tophat (v 2.0.8) using default options. Reads that failed to map into this genome were isolated and analyzed separately (see Section Differential Expression Analysis).

**Figure 1:**
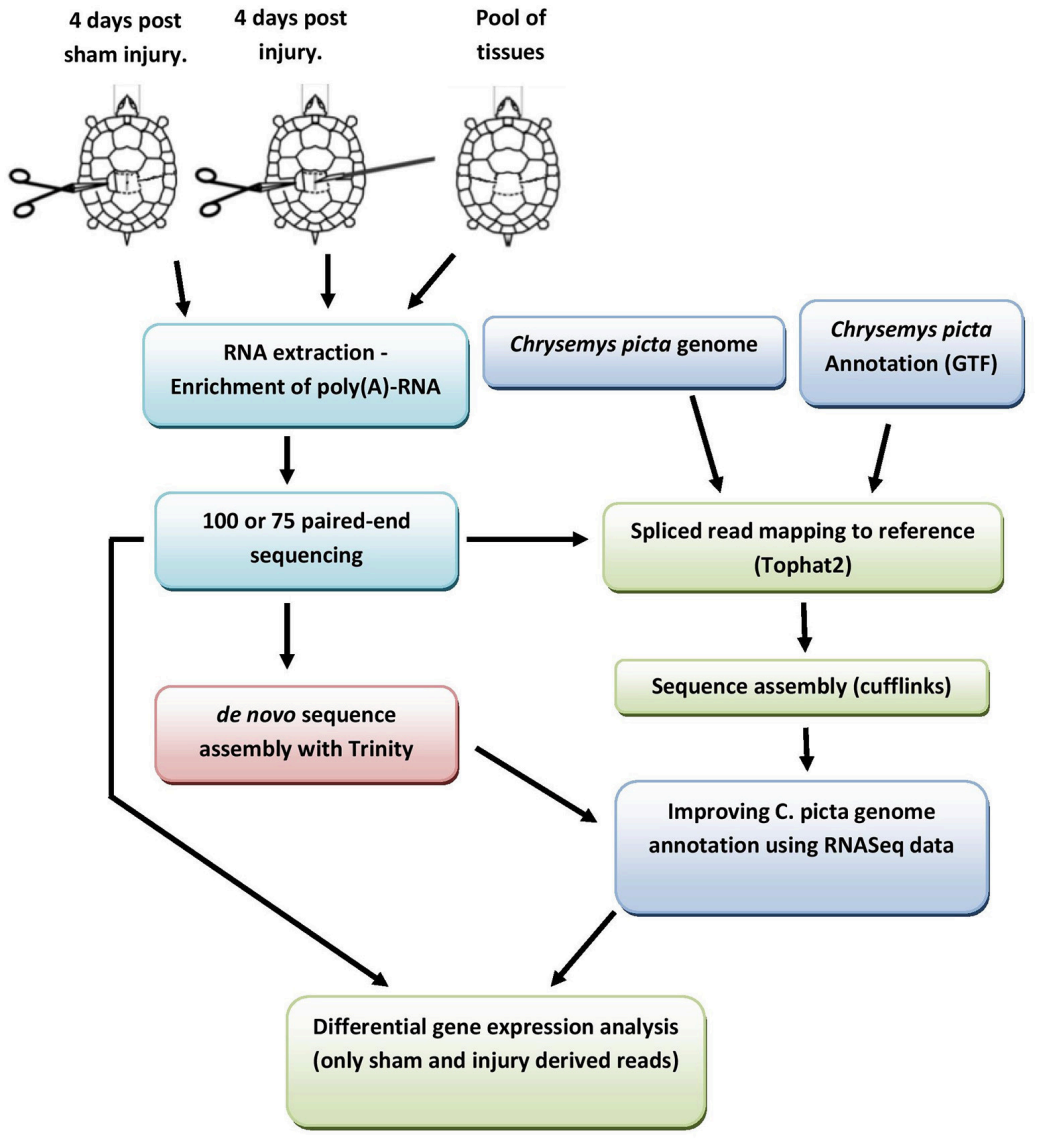
**Schematic depiction of the experimental pipeline used to generate an “enriched” ***C. picta*** mapping reference and subsequent differential gene expression analysis**. Further details in section under the title “*de novo* transcriptome assembly” and Figures S1–S6 in supplementary file.

Mapped reads were assembled using RBTA (Reference Based Transcriptome Assembler) with Cufflinks (Trapnell et al., 2012; v 2.1.1). Assemblies for each condition were combined with the *C. picta* reference genome (NCBI *C. picta* reference v3.0.1) using Cuffmerge (Trapnell et al., 2012; v 1.0.0) to produce a final enriched consensus genomic reference file (gtf file) that was used for downstream analyses (for details see Figure 1 and Figure SF4).

#### *De novo* transcriptome assembly

Only Illumina GAIIX reads (2 × 100 pe) were used for assembling. These were quality filtered: undetermined and low quality bases were trimmed out, and the resulting read fragments shorter than 70 nt were discarded (results are summarized in Figure SF1 and Table S1).

*De novo* assembly was performed using Trinity RNA-seq assembler (v r2013-05-25) which in the case of our dataset, outperformed other assembling algorithms tested (Abyss, Oases, and a two-step strategy combining Abyss and Cap3 as described in Supplementary File, in section under the title “*de novo* transcriptome Assembly” and Figure SF2).

The experimental pipeline of the analysis is depicted in Figure 1.

#### Differential expression analysis

TopHat2 (Trapnell et al., 2009) was used to map reads on the enriched *C. picta* mapping reference obtained with Cuffmerge as described in Section Improving the Annotation of *C. picta* (Reference) Genome Using RNAseq Data (i.e., *C. picta* genome, gtf enriched annotation file). When assessing the expression levels of divergent genes (or genes not present in the *C. picta* genome) using the “*de novo*” mapping reference, read mapping was done with Bowtie2 (Langmead and Salzberg, 2012).

Read counts were obtained with HTSeq-count (v 0.6.0) and differential expression analysis was conducted using DEseq2 (Anders and Huber, 2010; Love et al., 2014). Genes were considered as differentially expressed (DE) when the three following conditions were met: a value of FDR lower than 0.1 (FDR is the False Discovery Rate, a correction of the *p*-value to account for multiple simultaneous tests); a fold change in transcript abundance of at least two (in either direction) and a minimum number of mapped reads in at least one of the conditions not lower than 50.

### Functional characterization of differentially expressed genes: gene ontology and metabolic pathways analyses

Because the annotation of *C. picta* genome publicly available has no Gene Ontology (GO) terms associated to its genes, we generated a GO annotation using Blast2go (v 2.7.0). Genes containing several isoforms may pose biases because these isoforms share GO terms, therefore leading to their overrepresentation. To avoid possible artifacts, isoforms were collapsed into their corresponding genes using a homemade pipeline. Gene Ontology enrichment analysis was done using the Fisher's exact test applying FDR correction for multiple tests. Terms with FDR < 0.05 were denoted as enriched and used in downstream analysis. Enriched terms were filtered to extract the most specific ones (i.e., those that have higher information content; defined as relative frequency of occurrence of the term in the protein annotation dataset under consideration, see Lord et al., 2003), using the filtering tool available in blast2go suite. Analysis of metabolic pathway enrichment was conducted using Panther Classification System (http://pantherdb.org/).

### Protein-protein interaction analysis

Interactions among the proteins encoded by differentially expressed genes were assessed using STRING (http://string-db.org). In order to simplify the network and highlight the most biologically relevant interactions, we excluded those interactions with a confidence score below 0.7. Clustering of the network was obtained using the Louvain algorithm (Blondel et al., 2008), implemented in the software Pajek (v 4.09). The resulting network was exported to Gephi (graph visualization and manipulation software; Bastian et al., 2009) to conduct additional analysis and obtain graphical representations.

## Results

### Mapping references: reference genome and divergent gene set

**Figure 2:**
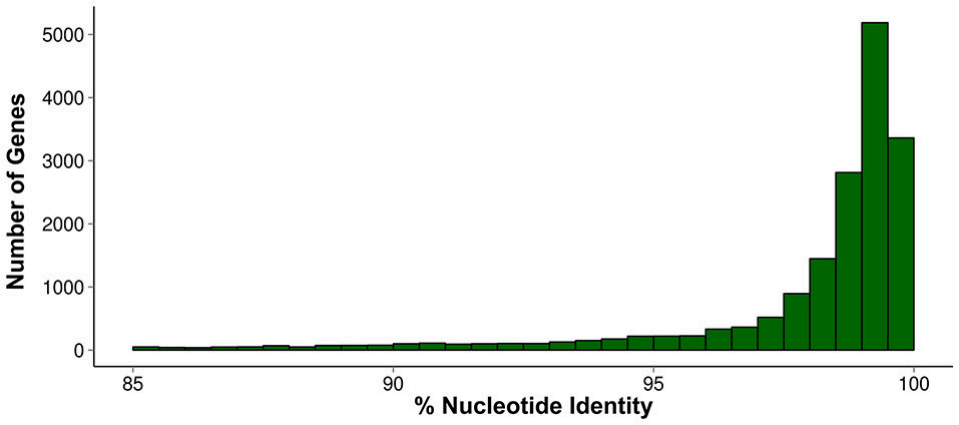
**Distribution of nucleotide identities between ***T. scripta*** and ***C. picta*** orthologous sequences**.

We used two complementary sources of data to obtain a reliable mapping reference: the assembled transcriptome from *T. scripta* and the genome sequence of *C. picta. De novo* transcriptome assembly was obtained using RNA isolated from three different tissues (liver, CNS, and muscle), in order to get a broad representation of the expressed genes as explained in Supplementary File. There are two complications when using the assembled transcriptome as the only mapping reference. One is that many low expressed genes often result in partially assembled mRNAs (or not assembled at all) due to their low transcriptional activity, even when the overall sequencing depth is high. Another complication is alternatively spliced mRNA as well as non-coding RNAs. The latter are often overlooked in RNAseq assemblies, hidden in the “jungle” of contigs resulting from this type of assemblies. Owing to these limitations we decided to complement RNAseq assembly with genomic sequence data from *C. picta*. Although, molecular phylogeny indicates that the two species are relatively close (Fritz et al., 2012), and therefore their sequences are expected to be similar, whether *C. picta* genomic sequences are similar enough to be reliably used as reference was checked. To this end, we estimated the genome-wide nucleotide divergence between the two species comparing the RNAseq assembly (RNA contigs) from *T. scripta* with the genomic sequence of *C. picta*. The comparison of 33,000 pairs of homologous (orthologous) sequences (distribution of nucleotide identities presented in Figure 2) shows that the majority of coding sequences (>90%), exhibit levels of nucleotide identity higher than 98%. This indicates that *C. picta* genome is largely appropriate to map *T. scripta* reads. Nevertheless, 8% of its sequences exhibit nucleotide identities that fall below of what it is acceptable for mapping, either over their whole span or in some delimited segments. This anticipates that a significant proportion of RNAseq reads will not map appropriately. It is worth noting that although it is reasonable to presume that *C. picta* could also heal its spinal cord (considering the close phylogenetic proximity), regeneration capability has not been assessed in this species.

RNAseq reads were mapped to the *C. picta* genome using TopHat. At this stage of the analysis this has a double purpose: first, to refine the annotation of *C. picta* genome and second to identify reads belonging to genes that are either *T. scripta* specific or divergent between these two turtle species. Around 60% of reads properly mapped to the *C. picta* genome. These reads were used to refine and increase the accuracy of *C. picta* genome annotation using Cufflinks pipeline (reconstructed transcripts were used to discover new splice junctions and thus finding novel transcribed sequences). This analysis shows that 94% of splice junctions identified by cufflinks were already reported in the *C. picta* genome, but 3% were partially novel corresponding to putative new isoforms and 3% were completely novel, which correspond to unannotated transcripts.

Reads not mapping to *C. picta* genome represent a substantial proportion as TopHat failed to map about 40% of them. This is somewhat surprising considering the degree of sequence similarity between *C. picta* and *T. scripta*. To analyze the possible origin of these unmapped reads, a randomly chosen subset (of 20,000 reads) was blasted (with blastx) against the nr database (Genbank) and with Blat against *C. picta* genome using more permissive matching requirements than those of Bowtie. We found that more than 90% of them had as their best hit turtle sequences (Figure SF3). This indicates that most of the unmapped reads are indeed *T. scripta* reads derived from protein coding genes. We conclude that these reads were unable to map to the *C. picta* genome because they may correspond to divergent genes, or divergent regions within genes, and the matching requirements imposed in the mapping were rather stringent (only 2 mismatches were allowed).

In order to use this piece of data, we isolated and assembled these reads to obtain a transcriptome of *T. scripta* divergent genes. The contigs obtained by this procedure could correspond either to complete fast evolving (divergent) genes or to divergent segments of otherwise conserved genes (for details see Figures SF5, SF6). To recover full length transcripts, we compared this assembly with the assembly derived from all reads; extracting the corresponding sequences from the latter, which most likely represent the full length transcripts (see Figure SF4). Finally, these “divergent” sequences were annotated by homology transfer of information.

### Differential expression analysis

Gene expression levels were found to be significantly different between control and 4 days after injury for 1057 genes. This figure, which is somewhat low when compared with what has been reported in other species, increases to 1300 if one uses a less restrictive criterion (FDR < 0.1, fold change > 1.8). Among the differentially expressed genes, 800 were over-expressed in the lesion whereas only 257 genes presented decreased transcription levels (see Map-plot in Figure SF7 and Table S2 contains the full gene list). The expression patterns exhibit high consistency within conditions (sham injured and injured animals) as it can be appreciated in the heat map (Figures SF8, SF9) which shows that for all genes the expression levels were more similar among replicates than between conditions.

### Functional analysis of genes exhibiting differential gene expression after SCI

As a first approach to understand the biological significance of the set of differentially expressed genes we conducted a Gene Ontology (GO) enrichment analysis. This yielded a large number of GO terms that are significantly overrepresented (Fisher's exact test, with correction for multiple tests by FDR < 0.05). To reduce the complexity and redundancy of this information, ontology terms were filtered using an information content criterion with the filter application included in blast2go (Conesa et al., 2005). Pathway enrichment analysis was also carried out as a complementary approach (since many of the proteins are enzymes) to help depict the functions involved in the SCI response. This was conducted using Panther (Thomas et al., 2003).

**Figure 3:**
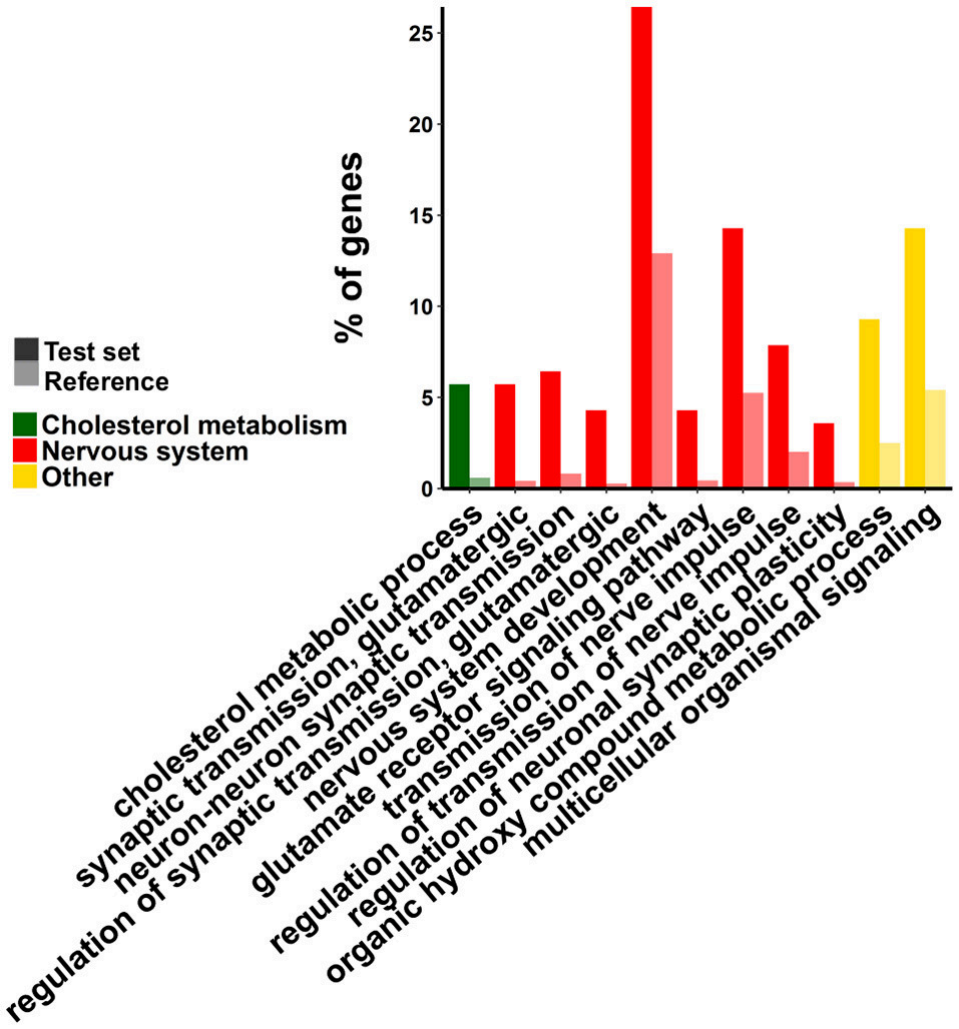
**Gene Ontology enrichment analysis of down-regulated genes**. Test set refers to the group of differentially expressed (DE) genes (in this case only down-regulated ones) and reference to all genes in the genome. Only some representative (and informative) terms were included in the figure.

The group of functions associated to down-regulated genes are schematized in Figure 3. Three main groups of GO categories can be readily identified: cholesterol biosynthesis, glutamatergic synaptic transmission, and other biosynthetic processes of small molecules. Detailed results are presented in Tables S3A–C. Enrichment analysis of metabolic pathways are in line with GO analysis since the pathways overrepresented are those related to the synthesis of cholesterol [P00014, genes Squalene monooxygenase (SQLE), Hydroxymethylglutaryl-CoA synthase (HMGCS1), FDFT1, and lanosterol synthase (LSS)], metabotropic glutamate receptor group I and III pathways (P00039 and P00041). This analysis gives further insight since it allowed us to identify other potentially relevant pathways which are overrepresented in the group of down-regulated genes as degradation of serotonin (P04372), cadherin (P00012), and Wnt (P00057) signaling pathways.

Concerning the set of genes with increased transcript levels, the scenery is far more complex since it includes hundreds of genes and GO categories. For the sake of simplicity, these GO categories were grouped into four main types: response to ischemic insult (hypoxia and oxygen reactive species), extracellular matrix re-organization, cell proliferation and death (mitosis, regulation of cell cycle, differentiation, organogenesis and apoptosis), and immune response and inflammation.

**Figure 4:**
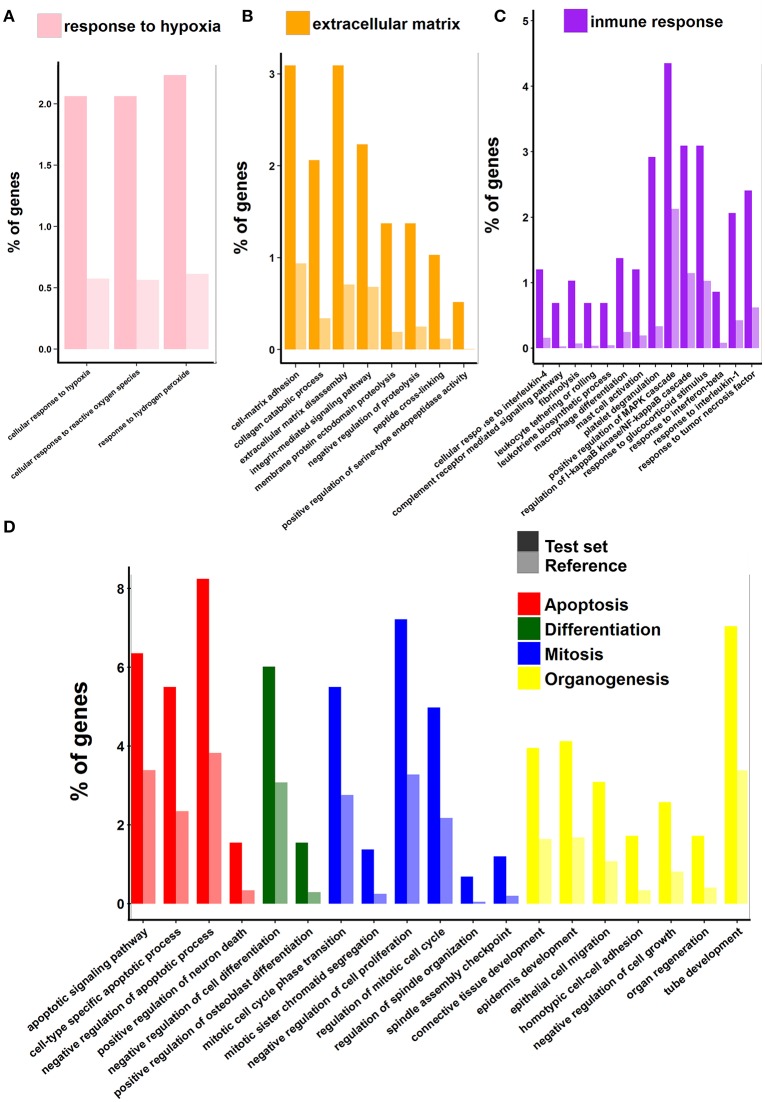
**Gene Ontology enrichment analysis of genes that are up-regulated**. Symbols as in Figure 3. Panels **(A–D)** show relevant groups of GO terms. The group of GO categories is indicated at the top of each panel.

The first group, related to hypoxia and reperfusion, contains genes encoding proteins that participate not only in the response to hypoxia but also in protective mechanisms against reactive oxygen species (Figure 4A, Table S3D). This is perhaps one the aspects of the SCI response that deserves more attention and is worth comparing with other vertebrates in greatest detail. The hypoxic ischemic insult, being caused by local destruction of vasculature, along with mechanical trauma, is one of the main causes of primary cell death. Hypoxia also triggers a cascade of biochemical and cellular responses (ischemic cascade) which promote inflammation (Van Elzen et al., 2008; Chadwick et al., 2011). Understanding the response to anoxia after SCI in freshwater turtles is particularly important because they are among the most tolerant organisms to oxygen deprivation. The CNS (brain) response to low oxygen level (or even complete anoxia) is remarkable and differs in many aspects to that of other vertebrates (see Nilsson and Lutz, 2004; Storey, 2007; Krivoruchko and Storey, 2015). This point is addressed in Section Response to Hypoxic-Ischemic Insult.

The second large group of genes and GO terms is that related to the extracellular space and the extracellular matrix (ECM) re-organization. This group comprises several GO terms (Figure 4B) which include more than 90 genes (Table S3E). Among these genes, those encoding matrix metalloproteinases (MMPs) and their inhibitors (TIMPs), desintegrins (ADAMs and ADAMTs), and cathepsins have been extensively studied before in other vertebrates and they were associated with a plethora of phenomena occurring after spinal injury (blood–spinal barrier integrity, inflammation, glial scar formation, axon guidance and regrowth, among others; Verslegers et al., 2013 and references therein). Other proteins and functions included in this group are: integrin-mediated signaling pathway (P00034), which links cell cytoskeleton to the extracellular matrix and senses whether adhesion has effectively occurred, blood coagulation (P00011), and dissolution of blood clots (P00050). The topic of extracellular matrix remodeling, the role of MMPs in SCI and the comparison of the behavior of these proteins between turtles and other vertebrates is discussed in Section Extracellular Matrix Reorganization Enzymes, Re-Vascularization and Axon Re-Growth.

The next group of GO terms and functions is particularly large containing more than 220 genes (listed in Table S3F). It comprises: cell death (apoptosis and its regulation), cell proliferation (mitosis, regulation of cell cycle), cell differentiation and organogenesis (gliogenesis, neural tube development, angiogenesis, epidermis development, epithelial cell migration, connective tissue development, regulation of osteoblast differentiation), and regulation of transcription (Figure 4D). Many of the functions included in this group are among the most preserved in vertebrates in terms of the genes that participate (see later).

The last group is composed by functional categories related to inflammation and innate and adaptive immune responses (Figure 4C). The group is also very populated (more than 180 genes, listed Table S3G) and complex. It comprises many crucial biological processes that take place during SCI response like: chemokine activation, complement system, regulation of leukocyte and macrophage migration, as well as antigen presentation.

Pathways enrichment analysis shows a complementary aspect to that presented in tables S3D–G. In effect, many of the genes falling in the groups of GO categories described before belong to metabolic pathways which are transversal, namely they touch several GO term groups. For instance, the CCKR signaling pathway (P06959), the p53 pathway (P00059), and the gonadotropin releasing hormone receptor pathway (P06664) are related to apoptosis, cell proliferation, and immune response. Other pathways like angiogenesis (P00005), JAK/STAT signaling pathway (P00038), interferon-gamma signaling pathway (P00035), and FAS signaling pathway (P00020) are shared among cell proliferation, differentiation, and immune response.

**Figure 5:**
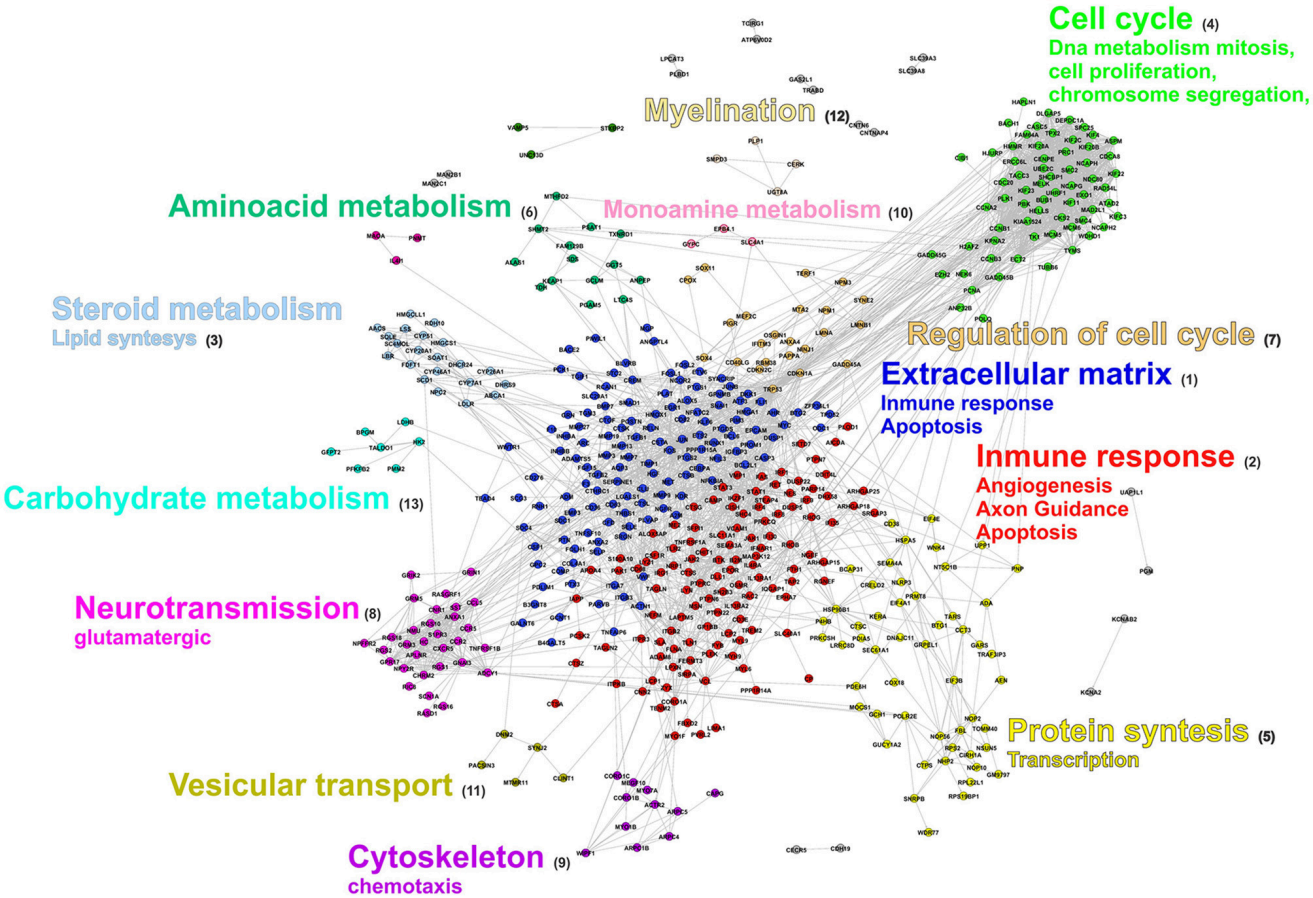
**Protein Interaction network of ***T. scripta*** DE genes**. Nodes represent genes and lines interactions. Node colors are used to identify the cluster to which the genes belong to. The most relevant cellular processes and functions associated to each cluster are specified by the legend next to it.

Finally, an integrative view of the biological characterization of the response to SCI was obtained by mapping the main GO terms (biological processes) just described over a network of protein-protein interactions. As shown in Figure 5 the main clusters correspond to clear functional groups. Yet some biological processes like apoptosis contain genes located in more than one cluster. The inspection of this network allowed us to identify some additional aspects. First, some clusters (relatively small) correspond to functional groups that did not clearly emerge in the GO and pathways enrichment analyses. The failure to detect them with the other approaches is precisely their small size, which prevents attaining high statistical significance. Examples of these are: carbohydrate catabolism (cluster 13), vesicular transport (cluster 11), and myelination (cluster 12). Second, although some proteins have functional annotation, they are not associated to any GO term or metabolic pathway, probably due to incompleteness of these databases. Many of these are members of clusters with evident functional characterization, meaning that they very likely are also involved in the function to which the cluster is related to.

In summary, the three approaches used to characterize the functional profile of the genes involved in SCI response complement each other showing different aspects of the response.

### Evolutionary distribution of DE genes

Since the ability of functional recovery after SCI is restricted to a few vertebrate groups, we could gain some insight on how this ability was gained (or lost) during evolution (and hopefully to identify candidate genes underlying this difference) by comparing the evolutionary distribution (presence/absence and degree of conservation) and variation in expression patterns of genes encoding proteins associated to regeneration. To this aim, we first determined the phylogenetic distribution of the group of differentially expressed genes. These genes were used to search for their corresponding orthologs in a collection of species that have different regenerating capacities and are representative of the vertebrate taxonomic diversity: human, mouse, gray short-tailed opossum (*Monodelphis domestica*), chicken (*Gallus gallus*), the green anole (*Anolis carolinensis*), *Xenopus tropicalis*, Zebrafish (*D. rerio*), and the Chinese soft shell turtle (*Pelodiscus sinensis)*. To identify orthologs we used a strategy that combined an automatic detection step using the program fastortho (http://enews.patricbrc.org/fastortho/) and online web tools containing databases of vertebrate orthologous genes. Additionally, for the case of genes having incomplete annotation in some species and/or conflicting evidence, other resources were used. Details of these procedures are explained in Supplementary File, section under the title “Orthology assignment.”

**Figure 6:**
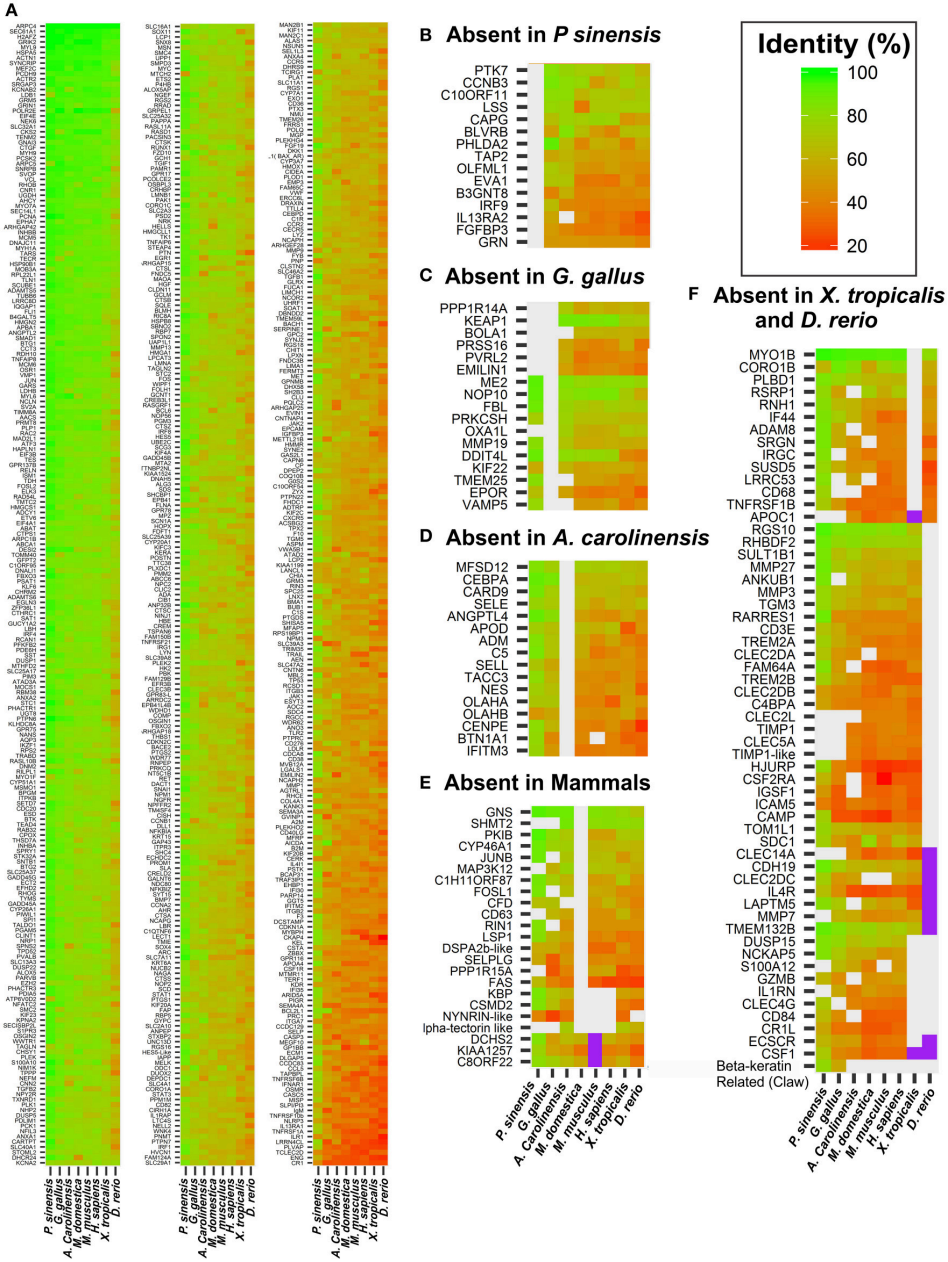
**Amino acid identity levels of DE genes in species representative of vertebrate taxonomic diversity**. Identity levels are indicated by the color scale shown in the top leftmost corner of figure. Values indicate the identity between *T. scripta* and the species indicated at the bottom of heatmap. Groups of genes for which no ortholog could be found in a given species are presented in separated panels. **(A)** Genes that are present all the vertebrate species used in this study. Panels **(B–F)** show genes absent in a particular species, or clade, as indicated in the sub-title of the respective panel. Genes for which an ortholog could not be unambiguously identified in a given species are indicated in violet.

Results from this analysis are illustrated in Figure 6. Most genes are present in all species (Figure 6A), exhibiting diverse degrees of conservation throughout evolution, whereas a few exhibit a scatter distribution being absent in some species (Figures 6B–F). These gene absences can be attributed to a number of reasons. In some cases this is most likely due to incomplete assembly and/or annotation. This is the case in the majority of the 32 genes that were not found in the soft shell turtle *Pelodiscus* (Figure 6B). In fact these genes are present in all vertebrates analyzed in this study, including the two other turtle species (*C. picta* and *T. scripta*). In addition, we checked their presence in another turtle species (the green sea turtle *Chelonia mydas*). With only few exceptions (BOLA1, CAPG, OXA1L, EMINIL1, and TIMP1), they are present in the genome of *C. mydas* and exhibit high sequence identity with their *Trachemys* orthologs (complete list in Table S4A), giving support to the notion that the failure to detect them in *Pelodiscus* is attributable in most cases to the incompleteness of its genome.

In other cases the absences can be attributed to lineage specific gene losses. This is the case of some genes associated with immune response, ossification, vasculogenesis, and neuron apoptosis that have been lost in Aves or green Anole (Figures 6C,D. Complete list in Table S4B), as well as three genes that were not found in any of the three mammalian species analyzed in this work (Figure 6E, Table S4C).

Two other interesting observations emerge from Figure 6. First, there are very few genes that are present in all (or most) amniotes but absent in both *Xenopus* and Zebrafish. Among these, some examples are: NCKAP5, S100A12, CR1L, DUSP15, ECSCR, and IL1RN. These are more probably amniote-specific gene acquisitions rather than losses in the two anamniote lineages. Additionally, there are around 20 genes associated with SCI response in turtles that are present in all tetrapod (including *Xenopus*), but absent in Zebrafish (Figure 6F, gene list in Table S4D). This group would represent tetrapod gene innovations, yet some genes might also be *Danio* lineage specific losses. The two possibilities were tested by using the genome sequences of two other fish species that are distant relatives: fugu (*Takifugu rubripes*) and medaka (*Oryza latipes*). The details of this analysis (presented in Table S4D) show that with 3 exceptions (FAM64A, RHBDF2, and CD3E) they are exclusive of tetrapods. Enrichment analysis of this gene group representing tetrapod innovations shows that it mainly consists of genes related to natural killer cell activation (GO:0030101, GO:0001819, GO:0034097), immune response (GO:0006955, GO:0045087), inflammation (GO:0006954), regulation of cytokine production (GO:0001819), and extracellular matrix remodeling (GO:0030198, GO:0030574, GO:0010951).

The second, and more relevant aspect, is the absence of a group of genes exclusive of regenerating taxa, namely shared only by Zebrafish and turtles. This is an important observation because together with the absence in *Danio* of about 20 genes associated with SCI in turtles, it suggests that the processes that lead to functional recovery in these two taxa probably rely on very different cellular/molecular mechanisms.

### Expressed sequences with no annotations

The construction of an improved mapping reference allowed us to identify 8389 transcribed genomic regions corresponding to previously not annotated sequences. To get insight on the nature of these sequences (coding, regulatory etc.) we conducted some basic analysis: we used the CNCI pipeline (coding non-coding index). A draft annotation of these sequences is presented in Table S6 (*de novo* transcript sequences). This analysis indicates that 211 sequences from this set could be classified as protein coding, although no one belongs to the set of differentially expressed genes. Besides, a blastn search shows that 566 of these sequences gave hits inside coding regions of other turtle genomes. A careful look reveals that these hits are located in the 3′ UTR and relatively far away from the stop codon (varying from 500 to 2000 bases from stop codon). We speculate these sequences could correspond either to genes or gene segments not annotated in *C. picta* genome, or to ncRNA which map on the 3′ side of the transcripts. The remaining ones were classified as unknown RNAs. It is probable that these sequences, despite not having lncRNA characteristics, may correspond to some kind of regulatory RNA or other type of functional RNA.

Out of the 1057 sequences that comprise the set of differentially expressed genes/sequences; 80 do not match to previously annotated protein coding genes of *C. picta*. Among them, 59 belong to the group described in the previous paragraph, namely regions in the *C. picta* genome that do not have annotations. Notably, none of them had protein coding features. The remaining 31 DE transcripts did not map into the *C. picta* genome but were detected in the “*de novo*” mapping reference (i.e., assembled transcriptome). Of these, three have homologous sequences in other turtles (e.g., *Pelodiscus* or *Chelonia*) and 28 did not yield any blast hit to turtle genomes.

### Conservation and divergence of expression patterns in genes involved in SCI response

Even though genes associated to SCI response in turtles are present in all (or most) amniotes, and the majority of them (>95%) in all vertebrates, they do not necessarily need to be related to SCI response in all taxa. Hence, another important aspect that must be considered is the conservation/divergence of their expression patterns in SCI, since the comparison of expression variation of orthologous genes may yield some relevant clues on the differences between regenerating and non-regenerating taxa. To conduct this analysis we used RNAseq data of SCI response from the mouse (a non-regenerative species, produced by Chen K. et al., 2013). Data from *Xenopus* correspond to those presented in Lee-Liu et al. (2014). This latter species is of experimental importance because it contains both regenerative (tadpole) and non-regenerative (post metamorphosis) stages. This enables the study of genes and mechanisms turned off in the non-regenerative stage that are probably common to non-regenerative vertebrates. Even if what it is observed in the tadpole of Xenopus may also be valid for other anamniotes, the generality of the conclusions should be taken cautiously (see later in Section Ancestrality and Evolution of Spinal Cord Regeneration Capacity). Regrettably, no comparable RNAseq data are available for salamanders or Zebrafish, which are able to regenerate when adults.

Two categories of genes were defined according to the variation in their expression patterns between the two species being compared: those that have a departing behavior and those that have a similar (or equivalent) behavior. A gene was considered to belong to the first category when the ortohologs in the two species being compared (i.e., turtle vs. mouse or turtle vs. *Xenopus*) change in opposite directions, or the gene is over or under-expressed in turtles but remains unaltered, or with very little variation, in the other species. Owing to differences in sequencing depth in the three species being compared, the criteria of what is considered “little variation” was rather flexible (<40% in fold change lesion/control). In addition, one should also take into account the issue of time in these comparisons since the intervals after injury when the samples were taken are not the same in all species (4 days after SCI in turtles; 2 and 7 days in mice, 2 and 6 days in *Xenopus*). Furthermore, the pace at which the post-injury reaction process takes place is not necessarily the same in all species, meaning that the same amount of days does not necessarily imply that the process has advanced to the same extent in the two species. Therefore, the time scale is a key element for the comparisons to be meaningful. Consequently, to avoid possible distortions from this source in selecting genes for the downstream analyses, we chose a conservative criterion for defining departing and similar behavior. Specifically, orthologous genes were considered to exhibit departing behavior when they differ between turtles and mice (or turtles vs. *Xenopus*) at all time points under comparison. Similarly, genes were regarded as displaying equivalent behavior when the coincidence is observed at both time points. The remaining (“ambiguous”) genes were excluded from the functional characterization analysis. Although, this safety criterion might render some false negatives, it ensures that the genes classified as behaving differently or equivalently are effectively so. Table 1 shows the number of genes falling in the “discrepant category” at 2, 7 days and both time-points in the comparisons turtles-mice and turtles-Xenopus. In the case of mice, although the majority of genes exhibit similar patterns in both species (58%), the number genes with divergent expression patterns is substantial (26%). In turn, the comparison between turtles and Xenopus shows two different situations, that involving the regenerative phase (tadpole, stage 50) and the non-regenerative stage (after metamorphosis, stage 66). Surprisingly, the differences are more pronounced with the regenerative phase. In fact, this stage exhibits a radically different pattern of expression when compared with the turtle, as the majority of genes display a departing behavior in both time points (60 and 70% genes, for 2 and 6 days respectively, being 41% at both time points). On the other hand, a coincident behavior was observed only in 13% of genes. In contrast, the non-regenerative stage of *Xenopus* shows patterns more similar to that of turtles and mice, particularly at 6D after SCI, where the proportion of genes with departing and coincident behaviors is similar to that of mouse (36%).

**Table 1:** **Expression comparison between Turtle and Mouse, ***Xenopus*** regenerative, or ***Xenopus*** non-regenerative**.

	**Differing behavior 2D^a^ (%)**	**Differing behavior 6D^a^ (%)**	**Differing behavior 2D and 6D^a^ (%)**	**Coincident behavior 2D and 6D^b^ (%)**	**Ambiguous^c^ (%)**
Mouse	33	34	26	58	16
*Xenopus* regenerative	61	78	47	13	40
*Xenopus* non-regenerative	59	36	31	37	32

a *Differing behavior as explained in the main text; i.e., DE in T. scripta and −0.4 < FC < 0.4 in the other species being compared or with DE in both species but in the opposite direction*.

b *Coincident refers to the fact the gene is DE in both species (and in the same direction)*.

c *Ambiguous refers to the situation when the behavior is coincident at a given time point but different in the other time point*.

Considering that *Trachemys* is capable of healing and functional recovery after SCI, at a first glance this result seems somewhat unexpected. Our initial expectation was that the gene expression changes relate to SCI response in the turtle should be more similar to those occurring in the regenerative stage of *Xenopus* after SCI. Therefore, these comparisons strongly suggest that the molecular mechanisms that lead to spinal cord regeneration in anamniotes, represented here by the tadpole of *Xenopus*, differ in several fundamental aspects from those in turtles. In fact, the response triggered by SCI in *Trachemys* exhibits much higher resemblance to that of non-regenerative vertebrates.

**Figure 7:**
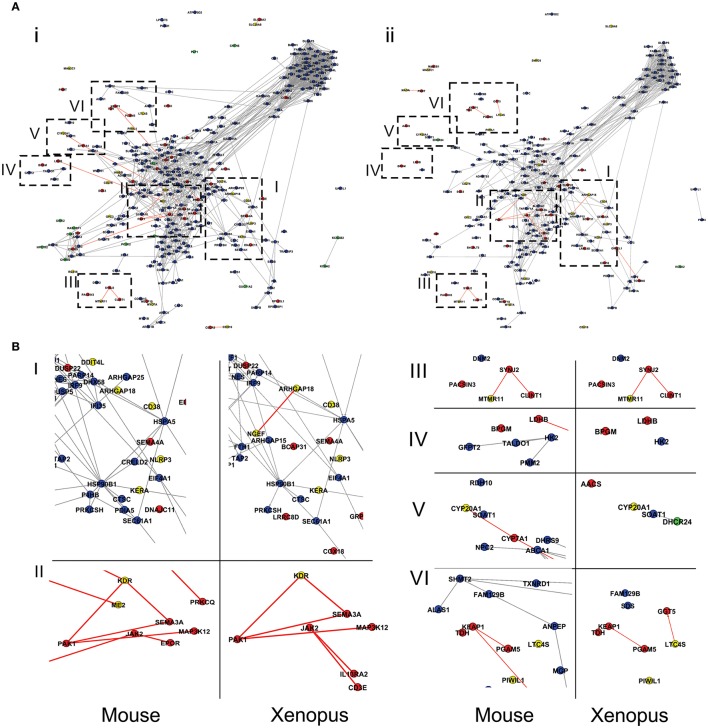
**Comparison of gene expression responses between turtles and mice (i) and turtles vs. NR-***Xenopus*** (ii)**. Panel **(A)** genes having double coincident behavior are colored in blue and green (for up- and down-regulated, respectively), whereas those exhibiting opposite responses are colored in red and yellow, for up- and down-regulated respectively. Further details about definitions on gene expression behavior in the text. Groups of genes of interest are boxed with dashed lines and shown in greater magnification in panel **(B)**.

The groups of genes having divergent and coincident behavior responses in the comparisons turtle-mouse and turtle-(nr) *Xenopus* were further analyzed to identify functions and cellular structures involved in each case. To this aim we identified the metabolic pathways and Gene Ontology categories to which the genes in these groups are associated (Table S5). Figure 7A shows, in the context of protein interactions network, the groups of genes having either double coincident (blue and green for up- and down-regulated, respectively) or double departing behavior (red and yellow for up- and down-regulated, respectively) in each one of these comparisons. Genes displaying ambiguous behavior were excluded in this analysis. As it emerges from the figure, although turtles appear to have a higher proportion of coincident genes with mice than with (nr) Xenopus, the genes that exhibit double discrepant behavior are roughly the same in both comparisons. The latter groups of genes are potentially very informative since they may provide insight on the differences of the molecular aspects of the SCI response between turtles and mice or turtles and non-regenerative stage of *Xenopus*. Among these groups of genes (presented in greater magnification in Figure 7B) we would like to highlight the following points: First, group IV including genes from the cluster associated to carbohydrate metabolism (cluster 13, Table S7). This group contains two genes BPGG (Bisphosphoglycerate mutase) and LDHB (L-lactate dehydrogenase B chain) which increase their transcript levels in turtles but remain unchanged in both mice and (nr)-*Xenopus*. Second, all but one gene belonging to group III, associated with vesicular transport (cluster 11, Table S7), show discrepant behavior between *T. scripta* and both mice and Xenopus. Other two groups that in our opinion are worth considering are: sector II containing a network of interconnected genes related to axon guidance mediated by semaphorins (e.g., SEMA3A, Semaphorin-3A, and PAK1, Serine/threonine-proteinkinase), angiogenesis (KDR Vascular endothelial growth factor receptor) as well as MAP3K12 (Mitogen-activated protein kinase 12), which is also associated with axon growth. Three of these genes are over-expressed in turtles (Sema3A, PAK1, and MAP3K12), but they are either under-regulated or remain unaltered in the two other species. In contrast, Vascular endothelial growth factor receptor (KDR) is strongly down-regulated in turtles, but is up-regulated in mice. Other genes, which do not belong to this cluster but are related to axonal regrowth (such as SEMA4A), are over-expressed in turtles but not in the other species. Finally, sector I (located in the region associated to immune response, i.e., cluster 2, Table S7), contains about 8 discordant genes, five of which are involved in cellular communication and localization.

In summary, the gene expression response observed in *Trachemys* is vastly more similar to that of mice and NR-Xenopus than to R-Xenopus. In addition, the genes that exhibit dissimilar behavior between turtles and the non-regenerating species compared here are basically the same in both comparisons. It is reasonable to postulate that precisely this latter groups of genes contains the most probable candidates to underlie the different SCI responses between turtles and mice and turtles and non-regenerative stage of Xenopus.

## Discussion

### Ancestrality and evolution of spinal cord regeneration capacity

In this study we describe the genetic response to SCI in freshwater turtles during the acute phase of the lesion. We found that this response involves more than 1000 genes affecting different metabolic pathways and biological processes. We analyzed the evolutionary distribution of these genes and found that with few exceptions most of them are present in all amniotes analyzed, and the missing ones being lineage specific losses. We did not identify any group of genes shared exclusively by regenerating taxa. This is in contrast with what was found in limb regeneration of salamanders, which is dependent of a retinoid-inducible gene, that encodes a three-finger protein called Prod1/CD59 (Diaz Quiroz and Echeverri, 2013; Geng et al., 2015). Therefore, we investigate whether the cord regeneration capacity of turtles could be related to gene regulation level. Specifically, we compared turtles expression patterns with those of mice and Xenopus (during both regenerative and non-regenerative stages), species for which equivalent expression data are available. Remarkably, we found that the response in turtles is more similar to that of mice and NR-Xenopus than to Xenopus during its regenerative stage. It is worth mentioning that although the conclusions that can be drawn from the comparisons involving the regenerative *Xenopus* stage cannot be automatically extended to all anamniotes, there are some indications suggesting that the mechanism of regeneration in frog larvae are comparable to those in salamanders. Indeed, the histological features (cell types involved and anatomy) of the cord healing process (Zukor et al., 2011; Gaete et al., 2012) as well as the characteristics of the inflammatory and immune responses that participate in the regeneration of limbs and spinal cord are similar in frog larvae and salamanders (see Section Cell Proliferation and the Formation of a Permissive Cellular Bridge).

Overall, the observations above mentioned indicate that the capacity for spinal cord healing and functional recovery in turtles does not represent the retention of an ancestral vertebrate feature lost in other amniotes, but most probably a turtle specific acquisition attained by re-organizing the expression patterns of genes shared by all amniotes. This inference is compatible with the modern phylogeny of reptiles, where turtles are placed as a sister group of crocodilians and birds instead of an early branching reptile lineage (Hedges and Poling, 1999; Zardoya and Meyer, 2001; Iwabe et al., 2005; Hedges, 2012). In this phylogenetic context, attributing the healing capacity of turtles the status of ancestral conserved character would imply that there were three independent losses of this capacity: in mammals, in squamata (lizards and snakes) and in the lineage leading to crocodilians and birds. This strengthens our previous conclusion that the ancestral regeneration capability (anamniota-like) was indeed absent in the amniotes ancestor.

### Response to Hypoxic-Ischemic Insult

The mechanical damage of tissues in the region surrounding the lesion produces destruction of blood vessels, which in turn generates ischemia in the lesion epicenter. Therefore, the injured zone experiences a drastic reduction of nutrients and oxygen for a relatively extended period of time. In addition, there is accumulation of catabolic products (lactate, ammonia, etc.) that usually have a toxic effect. However, turtles are peculiar in this respect because they are extremely tolerant to anoxia. Indeed, in many turtle species, the CNS and other sensitive organs (like the heart) can stand complete oxygen deprivation for more than 1 day at room temperature (and for months in freezing water) without suffering noticeable damage (Jackson, 2002; Lutz et al., 2003; Krivoruchko and Storey, 2015). This tolerance to anoxia is likely to play a significant role in SCI by preventing or reducing the initial damage caused by ischemia, as well as in the subsequent biochemical cascades triggered by oxygen absence which promotes further damage.

In mammals the response to oxygen deficiency is complex, involving the combined interplay of many biochemical, genetic, and physiological processes (Van Elzen et al., 2008). Briefly, it implies an increase of glycolytic ATP production and consequently the rise of acidosis (due to lactic acid production), which in turn produces additional negative effects such as protein denaturation. Neurons and glial cells exhibit a highly detrimental loss of ionic homeostasis, which is caused by deficient functioning of membrane channels (owing to ATP scarcity). Membrane depolarization stimulates the release of excitatory amino acids, especially glutamate (glutamate excitotoxicity) which promotes further ionic alterations, in particular an exacerbation of intracellular calcium rise (Sánchez Alvarado and Tsonis, 2006). The molecular changes induced by these harmful alterations involve the induction of several cytoprotective pathways: the HIF (Hypoxia Inducible Factors, activated by HIF1-alpha) include more than 100 target genes related to the following processes: eritropoyesis, angiogenesis, cell proliferation, glucose transport, glycolysis, and free radical scavengers.

The response of turtles to anoxia involves several components, some of which are shared with mammals. A crucial aspect is their capacity to depress the metabolic rate to only 10–20% of the corresponding aerobic rate and enter in a nearly comatose state (heart rate falls 80%) when the whole body is in anoxic conditions. Neurons are virtually “turned off” in a process called channel arrest (Pék and Lutz, 1997; Pamenter and Buck, 2008). As a result, the decreased ion flux helps energy conservation through the reduction of ATP-demanding ion pumping, thus diminishing the negative side effects of excessive anaerobic fermentation. Whether these animals also elicit a similar response locally, namely a spatially restricted (to the injured region) channel arrest, remains to be determined. The results presented in Table S7 and Figure 5 (clusters 8) support this possibility. Specifically, genes encoding proteins related to neurotransmission such as the glutamate ionotropic receptors NMDA 1 (GRIN1) and kainate 2 (GRIK2); gutamate metabotropic receptors 3 and 5 (GRM3, GRM5) are strongly under-regulated. Furthermore, other receptors and proteins related to neurotransmission like Neuromedin-U (NMU), acetylcholine receptor M2 (CHRM2), Neuropeptide FF receptor 2 (NPFFR2), cannabinoid receptor 1 (CNR1), and the sodium channel SCN1A are also down-regulated. Down-regulation of several glutamate receptors after SCI, has also been reported in mice (Chen K. et al., 2013). However, this observation was interpreted by these authors as the consequence of neuronal cell death rather than as a cell protective response to avoid glutamate excitotoxicity. Like in mammals, another key element in the response of turtles to anoxia is the re-organization of energetic metabolism. In aerobic conditions, ATP can be generated from several sources (carbohydrates, lipids, and amino acids), but in anoxia anaerobic glycolysis is the only way of ATP production. It has been observed in the liver and kidneys of turtles that some glycolytic enzymes are activated by ChREBP (carbohydrate responsive element binding protein), a transcription factor that is hypoxia responsive (Krivoruchko and Storey, 2014). The results presented in Figure 5 (cluster 13), show that in the injured cord of *T. scripta* carbohydrate metabolism is indeed activated. Interestingly, some of the genes up-regulated in turtles, also increase in mice after SCI (Figure 7B, group IV). There are, however, two genes related to glycolysis, B- subunit of lactate dehydrogenase (LDHB) and Bisphosphoglycerate Mutase (BPGM), which are increased in turtles but remain unaltered in both mice and Xenopus. LDHB activity has been already reported to be anoxia responsive in turtles (*T. scripta*), yet in the liver but not in brain (Krivoruchko and Storey, 2015). This increase, however, is achieved by post-translational modifications that change its Km rather than by changes in the expression status of the gene (Krivoruchko and Storey, 2015). As for BPGM, the significance of its increased mRNA abundance is unclear. This enzyme is normally expressed in erythrocytes because it requires hemoglobin for efficient catalysis (unlike its paralog, the regular glycolytic enzyme Phosphoglycerate Mutase). The augmented mRNA level of this enzyme could be a local response of erythrocytes (which are nucleated in turtles) to anoxia. Alternatively, this enzyme might be expressed in neural cells and would interact with neuroglobin (a member of the globin family). Under anoxia neuroglobin is over-expressed in the CNS of turtles (Milton et al., 2006; Nayak et al., 2009) and other hypoxia tolerant vertebrates, but not in normoxic mammals (Avivi et al., 2010). It would be interesting to investigate whether BPGM has some kind of interaction with neuroglobin.

Since anaerobic glycolysis is not efficient due to the absence of oxidative phosphorylation, turtles contain large reserves of glycogen in the liver, which provide sugar for glycolytic ATP production. Turtles also have mechanisms for buffering glycolytic-caused acidosis. They release calcium carbonate from bones and shell and can store lactate in the shell (Jackson, 2002). Nevertheless, the relevance of these protective mechanisms is not apparent in ischemic anoxia, like that of SCI, because the insult is highly localized affecting a small proportion of the body. Consequently, large carbohydrate reserves do not seem to be necessary and the requirement of keeping acidosis at low levels on a very local scale is not nearly as demanding.

The differences between whole body and ischemic anoxia generated by SCI also become manifest in another critical biological process: cell proliferation. Cell cycle arrest is another component of the turtle response to (whole body) anoxia. But it is evident that such strategy can be hardly incorporated in a regenerative process because healing tissues require cell proliferation. In fact, our results show that one of the largest groups of genes is that encoding proteins related to cell division (clusters 4 and 7 in Figure 5 which include mitosis, DNA replication, and its regulation). The significance of cell proliferation and its role in SCI response is discussed in Section Cell Proliferation and the Formation of a Permissive Cellular Bridge. Therefore, delimiting the relative contribution of anoxia protective mechanisms and how anoxia tolerance is integrated in the response of turtles to SCI would be valuable to understand how these animals have acquired regeneration capacity. In this regard it is worth mentioning that Shaffer et al. (2013) have used RNAseq data to identify which genes are activated in the anoxic brain of *C. picta* (24 h of oxygen deprivation). They found 19 genes with substantial increase in transcript levels. Comparing this gene set with those reported here, reveals that although some are concordant, others are not activated in the injured spinal cord (FOSB, NR4A1, DDIT4) and several display marginal increase (EGR1, BTG1, BTG2, CYR61, DUSP1). Besides, a potentially valuable source of information to appreciate the incidence of anoxia tolerance in SCI would be to investigate the cord healing capacity in the Chinese softshell turtle (*Pelodiscus*) because anoxia tolerance in this species is only one-sixth of that in *T. scripta* or *C. picta* (Ultsch et al., 1984).

### Extracellular matrix reorganization enzymes, re-vascularization, and axon re-growth

More than 90 proteins that participate in extracellular matrix remodeling like MMPs and their inhibitors (TIMPs), desintegrins (ADAMs and ADAMTs), cathepsins and others are up-regulated in the acute phase of the response to SCI in turtles. These proteins and the genes encoding them have been extensively studied due to their importance not only in pathological processes but also in development and normal tissue remodeling (Rivera et al., 2010). The MMP protein family, composed in mammals by more than 20 members, is the most studied one. Some members from this family play dual opposing roles in SCI. Whether their role is beneficial or detrimental largely depends on time and context. Most studies were centered on gelatinases MMP9 and MMP2, which in mice are respectively over-expressed 2 and 7 days after cord injury. Their enzymatic activities were reported to be detrimental in the acute phase of injury because they degrade proteins from the basal lamina (laminin, fibronectin heparin sulfate) and tight junctions (VE-cadherim, occludin, zonulae occludens-1). This proteolytic activity promotes the loss of blood–spinal cord barrier integrity, allowing the infiltration of blood cells associated with inflammatory response and myelin degradation (Noble et al., 2002). Compelling evidence of a negative role for these proteins is provided by the fact that mutant (null) mice for MMP9 exhibit improved functional recovery compared with wild-type animals (Noble et al., 2002). Similar results were observed when these enzymes were pharmacologically inhibited (Zhang et al., 2011). The results presented herein show that turtles, like mammals, increase the transcript levels of MMP9 and other six MMP genes. However, in turtles MMP2 levels remain unaltered. In mammals MMP-12 (macrophage metalloelastase) is the member of the family that experiences the highest increase after SCI (120-fold increment) and was also associated with detrimental effects during the acute phase of the lesion, contributing, like gelatinases, to blood-CNS barrier disintegration (Wells et al., 2003). Likewise, MMP-12 null mice also attain higher recovery levels than WT controls (Wells et al., 2003). Importantly, the gene encoding this protein is not present in the *C. picta* genome and we could determine that this absence cannot be attributed to sub-annotation or incomplete assembly as the gene is also lacking in other turtle genomes. In other words, although the expression pattern of MMPs in the early SCI response of turtles has a considerable degree of overlap with that of mice, it displays marked differences in critical genes.

While the action of MMPs appears to be detrimental during the early phase of SCI, many of these enzymes could play beneficial roles in later stages. Three of such roles are: relationship with formation/dissolving of the glial scar, re-vascularization, and axon outgrowth/guidance. In mammals, after the acute phase of the lesion, a secondary injury response drastically limits the regenerative capacities. Reactive astrocytes start generating the glial scar (Fawcett and Asher, 1999), a special tissue conformation integrated by astrocytes themselves and a dense extracellular matrix (perineuronal net) predominantly composed by chondroitin sulfate proteoglycans (CSPGs). Although, axons are capable of certain amount of regrowth, the glial scar becomes an impenetrable physical barrier for outgrowing axons. In addition, astrocytes secrete diverse growth-inhibitory molecules which further inhibit axonal growth. Gelatinases (particularly MMP2) were proposed to play an important role in limiting glial scar formation by proteolytic degradation of CSPGs and the other inhibitory molecules (McKeon et al., 1991) thus clearing the route for axons (Veeravalli et al., 2009). Turtles, however, do not form a glial scar although evidence indicates that astrocytes (GFAP+ cells) are present at the lesion site (Rehermann et al., 2009). It would be of interest to determine whether this is due to higher and/or different MMP activity or to a different pattern of CSPG production by turtle astrocytes. Our results show that by day 4 (what would represent the end of the acute phase) no signs of CSPG genes activation can be detected, but reactive astrocyte markers (GFAP) have already become very abundant (FC = 2, see also García et al., 2012).

MMP enzymes have been suggested to promote axonal regrowth not only by their participation in clearing the advancing axonal pathways but also regulating other mechanisms. MMP9 is found in the axoplasm of myelinated regenerating fibers in peripheral nervous system (Kim et al., 2012) playing regulatory activities related to neurite extension and axon elongation (Shubayev and Myers, 2002, 2004). In this regard it is worth considering a circuit of potentially relevant genes linked to gelatinases. Specifically, our results show a group of four genes that interact with each other and have opposite expression patterns in turtles in comparison to *Xenopus* and mice. These are Sema3A, KDR, MAP3K12, and PAK1 (Figure 7B, sector II). Semaphorins (Sema3a) act as chemorepulsive molecules in axonal guidance processes and as signaling molecules in re-vascularization (Mecollari et al., 2014). KDR (also known as VEGFR-2) is responsive to the vascular endothelial growth factor (VEGF) and could be regulated by PAK1 (Bagheri-Yarmand et al., 2000; Birukova et al., 2009). VEGF mRNA abundance is also increased in injured turtle cord, yet to a lower degree than the other genes, being the values at the limit of significance (FC = 1.8, FDR < 0.1). This group of genes can be under control of gelatinases as in the case of testosterone induced mechanism of angiogenesis and neuronal recruitment, observed in the songbird forebrain, where MMP-2 triggers the cascade activating VEGF by post-translational modifications (Chen Z. et al., 2013). These genes also play dual roles (vascularization and neurite outgrowth) in rodents, with VEGF interacting with MMP enzymes (Rosenstein et al., 2010). Indeed, several authors reported that the trio VEGF/KDR/Sema3A promotes neurite outgrowth in the adult brain and during development (Rosenstein et al., 2003).

Evidence implicating these genes in mammal's SCI is more elusive. On the one hand, it has been shown that in mice VEGF increases angiogenesis potential after SCI improving the outcome (Facchiano et al., 2002; Widenfalk et al., 2003; Herrera et al., 2009; Patel et al., 2009; Sundberg et al., 2011). In contrast, Sema3A activity seems to be detrimental, acting as inhibitor of axon growth (Hashimoto et al., 2004). In fact, the use of xanthofulvin, a pharmacological inhibitor of Sema3A, has been proposed as a therapeutic strategy (Kaneko et al., 2006). In summary, the specific and prompt activation of this molecular circuit in the injured spinal cord of turtles could contribute to the quick reestablishment of brain blood barrier and growth of new vessels. Alternatively, taking into consideration that KDR (VEGF receptor) is strongly under-regulated in turtles, it is not expected to act as a mediator between VEGF and the other members of the group. This is highly suggestive that the mechanisms of activation of this group of genes probably differ from those of mammals. It is thus reasonable to raise the question of whether the role in SCI response of this gene circuit might have also changed between turtles and mammals. A likely alternative function may be its association with early activation of axonal regrowth mechanisms. In this sense it is worth taking into account that in *T. scripta* this process starts already by day 6–7 post injury, as evidenced by the fact that growing axons can be observed 7 days after injury (Rehermann et al., 2009).

It is interesting to note that these genes are downstream effectors of Notch1, a gene that has been proposed to play opposite roles after injury: a positive regulatory role in regenerating organisms (Dias et al., 2012) but a detrimental one in non-regenerative vertebrates where it promotes astrogliosis that leads to glial scar formation and inhibits axon regeneration (LeComte et al., 2015). Nevertheless, NOTCH1 was not detected as up-regulated in turtles. Yet, we found other genes that also are under the regulation of Notch1 to be over-expressed in turtles (HES5, STAT3, MYC, and MMP9). Taken together, these data would indicate that very likely Notch1 experienced a transient increase some time before the time point we are analyzing here, and hence we are detecting the effects of its earlier expression. This is in line with previous studies that reported that this gene may exhibit a brief pulse of increase after injury triggering cascades of regulatory events that promote glial differentiation and the loss of neurogenic capacity (Morrison et al., 2000).

### Cell proliferation and the formation of a permissive cellular bridge

The formation of a cellular bridge permissive for the growth of regenerating axons is a common strategy of species with regenerating capabilities and involves critical cellular processes such as proliferation, migration, and differentiation. In the earliest stages of repair, proliferation of neural progenitors, astrocytes, and other types of glia (e.g., oligodendrocyte progenitors), microglia and endothelial cells for angiogenesis dominates the scenario in both, non-regenerating (Velardo et al., 2004; Chen K. et al., 2013; Lee-Liu et al., 2014) and regenerating species (Chevallier et al., 2004; Hui et al., 2014; Lee-Liu et al., 2014). Our results show that also in turtles a large number of up-regulated genes (234) are related to these processes (Figure 5 and Table S7). The genes involved in proliferation and its regulation form two large interconnected clusters (cluster 4 and 7 in Figure 5) that are basically conserved in other vertebrates, namely genes belonging to these groups exhibit similar expression patterns in turtles, *Xenopus* and mouse (Figure 7).

In other regenerative species, the scene is similar. For example in Zebrafish the proliferative response to injury in the spinal cord is controlled by many cell cycle genes and other regulators of proliferation, with some genes common to other regenerating tissues like the fins, retina, and the heart (Hui et al., 2014). STAT3 pathway, which controls proliferation is activated at 1 and 3 days post injury (dpi) in Zebrafish. This pathway is also related to peripheral nervous system regeneration and regulates SOCS3, ATF3, MMP9, and SOX11. STAT3 co-localizes with both glial and neuronal markers, suggesting it is involved in neural proliferation. In turtles (this study) these genes are up regulated 4 days after injury, as they are in mice and non-regenerating *Xenopus* (Figure 7).

Concerning cell cycle control, we found over-expression of cyclin CCNA2, (essential for G1/S and G2/M transition phase), CCNB3 and cyclin regulators CDKN1A, and CDKN2B. In Zebrafish genes involved in the G1-S phase transition are up-regulated from day 3 to day 7 after injury but genes associated with the S phase are elevated only at day 7, when proliferation peaks. Ten out of twelve genes involved in the G2-M transition are up-regulated at 7 dpi (Hui et al., 2014). Similar changes in gene expression profiles take place in rats, in which by day 3 post injury (dpi) there is a shift from a damage control transcriptional profile to transcripts involved in proliferation and migration (Velardo et al., 2004). This shift has been proposed to be driven by hypoxia-responsive transcripts (e.g., hypoxia inducible factor-1 α) which regulate downstream genes that promote the proliferation of immune, mesenchymal, and angiogenic cells (Velardo et al., 2004). Interestingly, several hypoxia responsive genes are also up-regulated in turtles, suggesting that the mechanisms orchestrating the proliferative response to injury are shared by turtles and mammals, and may be general for vertebrate species.

Very recently Mokalled et al. (2016) reported that the connective tissue growth factor (CTGFA) would play a determinant role in the regeneration process of the spinal cord in Zebrafish. It was found that this gene is over-expressed 2 weeks after injury in glial and other cells types around the lesion where it appears to participate in the formation of a permissive cellular (glial) bridge. These authors show that loss of function mutations in this gene result in diminished glial bridge formation capacity. Conversely, over-expression of the gene or using local delivery of the human CTGFA leads to accelerated glial bridging, regeneration and functional recovery. Interestingly, we found that in turtles CTGFA experiences a three-fold increase during the response to SCI. However, neither the results reported by Mokalled et al. (2016) nor those presented here allow one to conclude that this gene could be considered a key player in determining the different outcomes observed in regenerative and non-regenerative vertebrates. The previous assertion is grounded on the fact that the data presented by Chen K. et al. (2013) show that the gene in question undergoes a big raise in transcript level also in (non-regenerating) mice, during both early-acute (2 dpi) and sub-acute (7 dpi) phases, reaching an almost 8-fold increase in mRNA level (in Table S2 from Chen K. et al., 2013).

### Inflammatory and immune responses

The inflammatory response has been proposed to be one of the determinants that preclude regeneration in mammals because it triggers the so called secondary insult. Moreover, the sole presence of immune cells in the CNS was considered pathological. In the injured spinal cord inflammatory response produces further destruction of neuronal and glial cells (expanding the damage) and eventually leads to glial scar formation (Goussev et al., 2003; Sánchez Alvarado and Tsonis, 2006; Horn et al., 2008). In line with this, much work aimed at decreasing the incidence of inflammatory response after SCI to prevent the formation of the glial scar has been done. In fact, for many years Methylprednisolone (a potent anti-inflammatory glucorticoid) was the only pharmacological treatment regularly used for acute SCI patients. Nevertheless, the efficacy of these treatments is currently under debate (Bowers et al., 2016). It was recently demonstrated that immunological signaling can confer important neuroprotective effects on the injured CNS (Gadani et al., 2015). In addition, other studies show that lesions generally worsen if some components of the inflammatory response are suppressed (Shechter et al., 2011).

Our results allowed us to identify the activation of many pathways related to inflammation and activation of innate and adaptive immune responses. Functional analysis of DE genes shows that a set of more than 170 genes, up-regulated in turtles 4 days after lesion, are related the activation of immune cells (leukocytes, mast cells, lymphocytes) and response to several inmunomediators (interleukins, and interferon beta gamma, complement). Among these genes, 92 are also up-regulated in mice and 67 in the non-regenerative stage of *Xenopus*. In contrast, in the regenerative *Xenopus* tadpoles genes related to the inflammatory and immune responses do not present evidences of activation after SCI (Lee-Liu et al., 2014). The RNAseq results obtained by these authors were confirmed using RT-PCR (Lee-Liu et al., 2014), giving further support to their transcriptomic findings. However, these results are in disagreement with previous analysis that reported the existence of an inflammatory response in *Xenopus* tadpole during tail regeneration (Love et al., 2013). In newts, in turn, inflammatory cells can be observed in the region of the transected cord (Zukor et al., 2011). Besides, genes associated to immune response and inflammation are up-regulated in Zebrafish after SCI (Hui et al., 2014). It is believed that inflammation inexorably leads to scarring and non-regenerative fibrotic wound healing in non-regenerative vertebrates, whereas in animals that can regenerate (Zebrafish, salamanders, and frog tadpoles) an inflammatory response does occur but is not detrimental to regeneration (Harty et al., 2003; Godwin and Rosenthal, 2014). In particular, *Xenopus* tadpoles lack of adaptive immune response which only appears after metamorphosis. The emergence of adult immune capabilities during maturation has been correlated with the loss of regeneration competence in adult *Xenopus* (Godwin and Rosenthal, 2014). Although, in salamanders there is an adaptive immune response, is very weak (Kaufman et al., 1995), as reflected by the fact that these animals are very sensitive to viral infections (Chen and Robert, 2011). Additionally, salamanders have a very slow cytotoxic immune response and the acute graft rejection reaction is missing (Koniski and Cohen, 1992). In other words, *Xenopus* tadpoles and salamanders have rather similar and somewhat weak inflammatory and immune responses, which appear to be compatible with regeneration instead to leading to fibrotic structural repair (Godwin and Rosenthal, 2014).

Our results show that turtles activate several components of the immune and inflammatory responses that would fall within the detrimental category according the above mentioned dichotomy, such as the activation of the complement system and the regulation of B and T cell proliferation, among others (see Table S3G). One fundamental element of early immune response in SCI is the activation of the complement system. Complement classical/alternative pathway and C3b/C4b activity inhibition after SCI decreases complement deposition and leukocyte infiltration, which is paralleled by increased tissue sparing and improved locomotor recovery (Reynolds et al., 2004; Jäderstad et al., 2010). We found 3 genes, C4BP4, CR2, and CD55, which are related to the activation of this pathway that are over-expressed in both turtles and non-regenerating species. On the other hand, blocking the high-affinity C5a receptor CD88 induces demyelination (Beck et al., 2010). Interestingly this pathway was not found to be activated after injury in turtles.

To conclude we want to emphasize that turtles exhibit a distinctive expression behavior for many genes, some of which appear to be meaningful in explaining the opposite outcomes observed when compared with mammals and adult *Xenopus*. However, the fact that the gene expression response in turtles is more similar to that of non-regenerating models than to *Xenopus* tadpoles, suggests that in spite of being capable of functional recovery, turtles display a more “non-regenerative-like” response. In this context, the differences in gene expression behavior observed for some genes between mice and *Xenopus* with turtles might be the key to regeneration in the latter. These genes are obvious candidates for further functional studies regarding new hypotheses on specific aspects of spinal cord regeneration. It is important to stress that the expression of several of these genes or the action of their encoded proteins can be pharmacologically manipulated (e.g., MMPs, VEGF, Sema3a etc.). Therefore, without being constrained by the requirement of conducting difficult genetic manipulations, turtles can also become an essential model for testing the specific role of some genes in the adaptive plasticity triggered by SCI, thereby contributing to the development of novel therapeutic strategies. Needless to say that the results presented in this work cannot be regarded as fully comprehensive, as they are restricted to 4 days after injury. Including additional time points will certainly result in a broader appreciation of the response's progression in turtles.

## Author contributions

AVK, GG, CR: conducted Molecular Biology experiments. AVK, FAV, RR, OT: wrote the paper. AVK, FAV: performed data analysis. FAV, CR, OT, RR: conceived the study.

## Funding

This work was partially supported by Comisión Sectorial de Investigación Científica (CSIC, Universidad de la República, Uruguay; grant number I+D-2014-327).

### Conflict of interest statement

The authors declare that the research was conducted in the absence of any commercial or financial relationships that could be construed as a potential conflict of interest. The reviewer JG and handling Editor declared their shared affiliation, and the handling Editor states that the process nevertheless met the standards of a fair and objective review.
