# Supplementary material for: Gene Expression Profiling in the Injured Spinal Cord of Trachemys scripta elegans: An Amniote with Self-Repair Capabilities
Source: Front Mol Neurosci. 2017 Feb 7;10:17. doi: 10.3389/fnmol.2017.00017 (PMC5293771; doi:10.3389/fnmol.2017.00017)
Supplement: Supplementary file 8 [file DataSheet1.pdf]

Supporting material for

Gene Expression Profiling in the Injured Spinal Cord of Trachemys scripta: An Amniote with Self-Repair Capabilities.

Valentin-Kahan et al., 2017.

De novo transcriptome assembly

Since no genome reference exists for *T. scripta*, a de novo assembly was carried out. Among the seven libraries sequenced in this study only that prepared using *T. scripta* RNA, isolated from a pool of three different tissues (liver, CNS, and muscle) was used in this phase of the analysis. Before assembling, sequencing reads obtained from this library (2x100 nt paired end, obtained with GAIIx) were quality filtered. Specifically, undetermined and low quality bases were trimmed out, and the resulting read fragments shorter than 70 nt were discarded. The read set thus obtained was subjected to further filtering in order to eliminate adapters and rRNA derived reads.

We tested 4 pipelines: Trans-abyss, Cap3 + Abyss, Oases and Trinity, aiming to determine which one performs better in our dataset, namely optimizing two parameters: the completeness of the reconstructed transcripts and the number of transcripts that are effectively recovered The test was conducted as follows: a group of 62 full length *T. scripta* mRNAs (downloaded from GenBank which were obtained by Sanger sequencing from individually isolated mRNA molecules) was used as the gold standard. For each of the assemblers we measured the number of mRNA that were recovered and the % of completeness. A Q scores, which was defined as the weighted averaged of the quantity and quality of reconstructed mRNAs ($Q=\sum NiPi$., where P is the proportion of the reference mRNA that is well reconstructed (i.e. completeness) and Ni is the number of mRNAs reconstructed at a Pi proportion, further details described in Greif et al, 2013). As shown in the figure presented below, Trinity was the assembler which performed the best. In effect, even if the combination of Abyss+CAP3 reconstructed a higher proportion of mRNAs, the completeness of its reconstruction are considerably lower than that of Trinity. In turn, Oases and Abyss(alone) clearly down-performed in comparison to the other two. This final assembly obtained by Trinity, and used for downstream analysis, consisted of 244912 contigs with a N50 of 1143 bp. The assembly was done using both paired-end and single reads (orphan pairs after filtering) as described by the developers of Trinity.

Orthology assignment

Identification of orthologs was performed using a strategy that combined an automatic detection step using the program fastortho (http://enews.patricbrc.org/fastortho/) after which we integrated other sources of information such inspection of phylogenetic trees and online web tools containing databases of vertebrate orthologous genes.

For fastortho program, the inputs were multifasta files containing all annotated genes (amino acid sequences) from each species. Fastortho uses blastp with a default e-value of 1e-5. From an initial group of homologous genes, fastortho attempts to distinguish between orthologs and paralogs using the Markov Cluster algorithm (MCL; Van Dongen 2000; http://micans.org/mcl/) in the same way as Orthomcl (Li, Stoeckert and Roos, Genome Res. 2003.). For powering the entries in the similarity matrix we used the default inflation value=1.5 (MCL, powers both the matrix and the individual entries).

The set of COGs (Clusters of Orthologous Groups) obtained by using fastortho was submitted to additional checks and refinements. First we addressed special situations; these are the cases of several genes that were missing in the genome of the turtle *Pelodiscus sinensis* that we suspected could be the result of incomplete genome assembly/annotation. This was done by searching for their presence in the genome of another turtle species (*Chelonia mydas*). The results of this study are presented in table S4a. Blastn, and tblastn were used to search in the genomes of *Gallus gallus* and *Anolis carolinensis*. Some genes we suspected their absence could also be attributed to incomplete assembly/annotation (for these two species we also used HCOP, see later).

We also checked in detail problematic COGs having inconsistencies and gene absences. The former refers to situations with higher or lower % of amino acid identity than what would be expected according to the phylogeny. For instance, turtles and human with significantly higher amino acid identity % than turtles and chicken. These "problematic" COGs, were analyzed by visual inspection of reciprocal hits in NCBI blastp and inspection of the phylogenetic tree built with the group of homologs (also examining the phylogenetic trees that are prebuilt in databases of homologs). In many cases the inconsistencies could be attributed to the fact that the annotation in one of the problematic species includes only a gene segment (having lower or higher conservation than the average of the gene). In other cases the inconsistencies were due to incorrect orthology assignment, and these were manually corrected. Other type of COGs considered “problematic” are those having gene absences. An important source of information for checking these latter COGs consisted in searching the presence of homologs in databases of othologous genes. This search was done on the basis of standard gene symbols (gene names). For this purpose it was necessary, in some cases, to annotate the *C. picta* sequence since had no gene symbol associated to them. To conduct databese searches we used HCOP (http://www.genenames.org/cgi-bin/hcop), which is a meta-database of orthologous genes. HCOP integrates data from different sources, including other databases of orthologs such as OrthoDB (http://www.orthodb.org/), OrthoMCL (http://orthomcl.org/orthomcl/), NCBI Homologene (https://www.ncbi.nlm.nih.gov/homologene), Ensembl, Phylomedb (http://phylomedb.org/) among others. HCOP also includes data from species specific databases (which contain manually curated information) such as Zebrafish (ZFIN http://zfin.org/), *Xenopus* (Xenbase, http://www.xenbase.org/), Chicken (Birdbase http://birdgenenames.org/), etc.

In a few cases there were conflicting results between the fastortho results (which in turn rely on blastp) obtained by us, and those indicated by HCOP. That is, our results indicated that a given gene was absent in a given species, nevertheless an otrtholog to the gene in question was reported in the database. Whenever these contradictions appeared we proceeded as follows: the gene was marked as uncertain in figure 6 (violet) and table S4, and we also used psi-blast, a more sensitive homology search program, to look for it in the corresponding virtual proteome. Another source of data used to search for missing genes consisted in examining the genomes of other species that are comparatively close to the species where the gene is missing (such as *C. midas* for the case of *P. sinesis*, rats in the case of mice).

All the information concerning the conflicting evidence is detailed in table S4 for each problematic gene.

Figure SF1

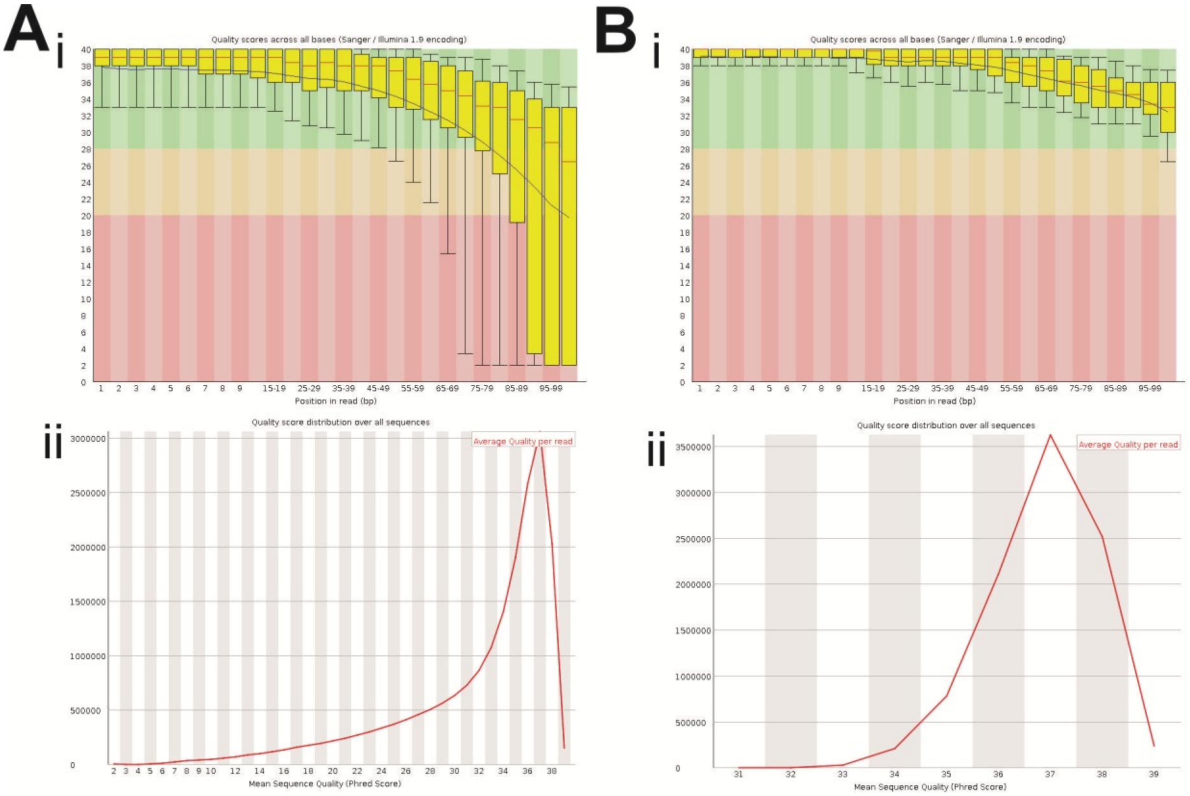

Figure SF1. RNAseq sequencing. A. Quality assessment of raw reads. Note the average lower base calling quality in the 3' region. B. Quality assessment of the same reads after filtering. Only bases with quality above 30 (1 error in 1000 bases) are kept for downstream analysis.

Figure SF2

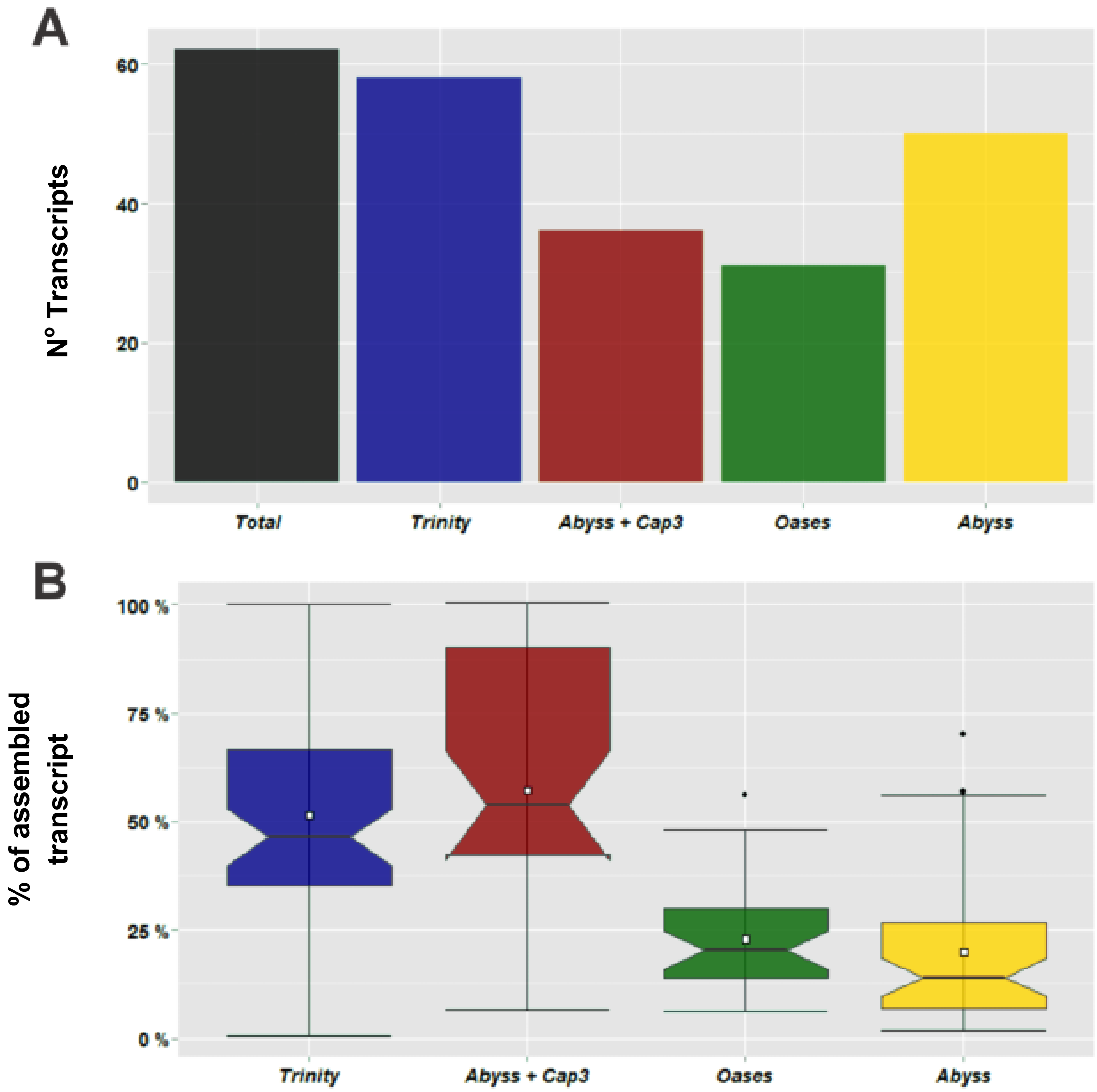

C

Programa	Q Score
Trinity	0.48
Abyss + Cap3	0.33
Oases	0.11
Abyss	0.16

Figure SF2. *T. scripta* transcriptome assembly. A. Number of known genes assembled compared to the total (black bar) for 4 assembly methods: Trinity, Trinity + Cap3 (denoted as Cap3), Oases and Abyss. B. Gene coverage of assembled transcripts. C. Q score (coverage * number of genes assembled) of each assembly method. Denote despite having less coverage the transcripts assembled by this method, it founds the great number of known genes. Known sequences were downloaded from NCBI.

Figure SF3

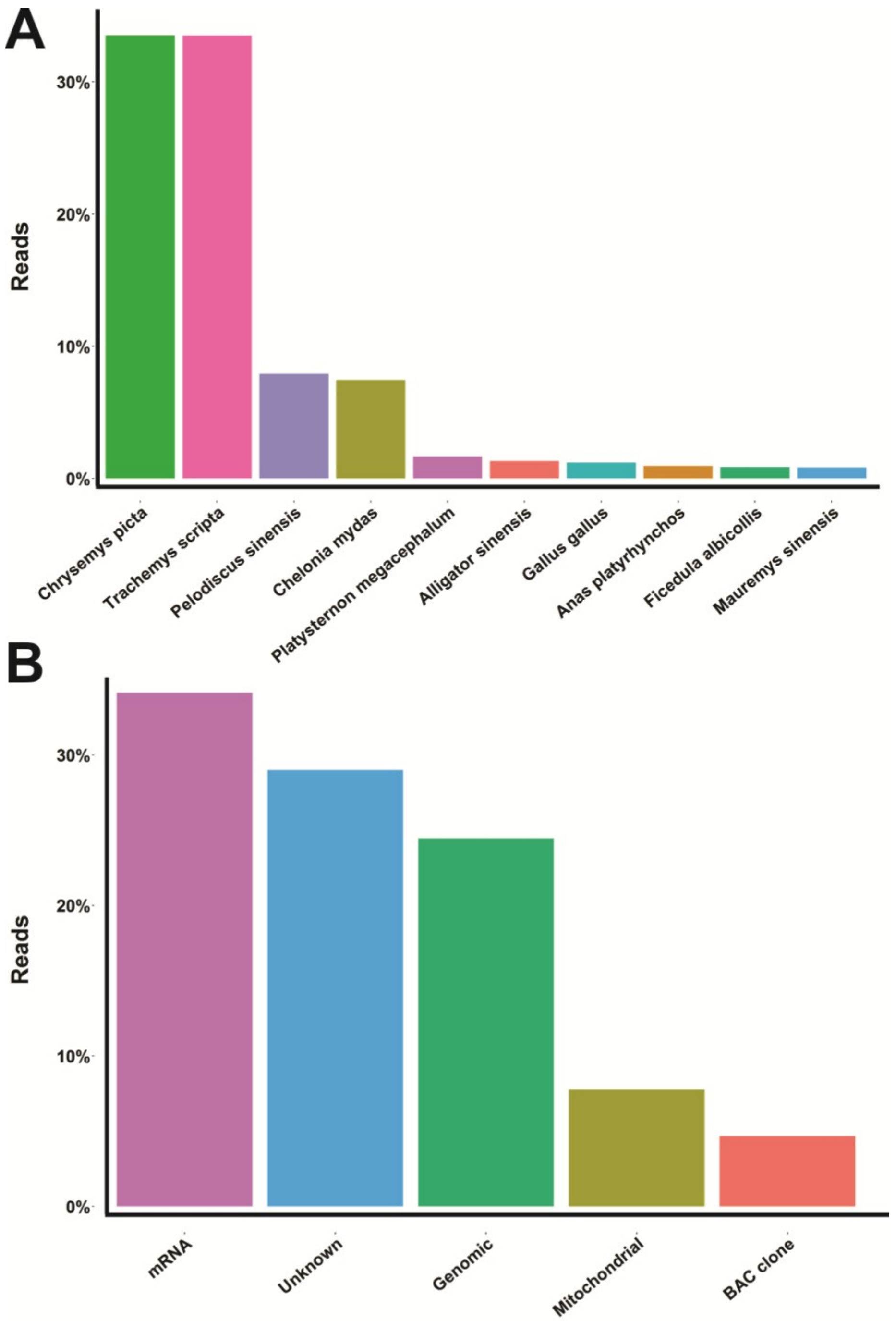

Figure SF3. Analysis of Unmapped reads on *C. picta* genome. A. Shows first hit of this reads to NCBI nr database. B. Shows potential origin of Unmapped reads. Genomic means reads mapping on non coding regions of any species.

Figure SF4

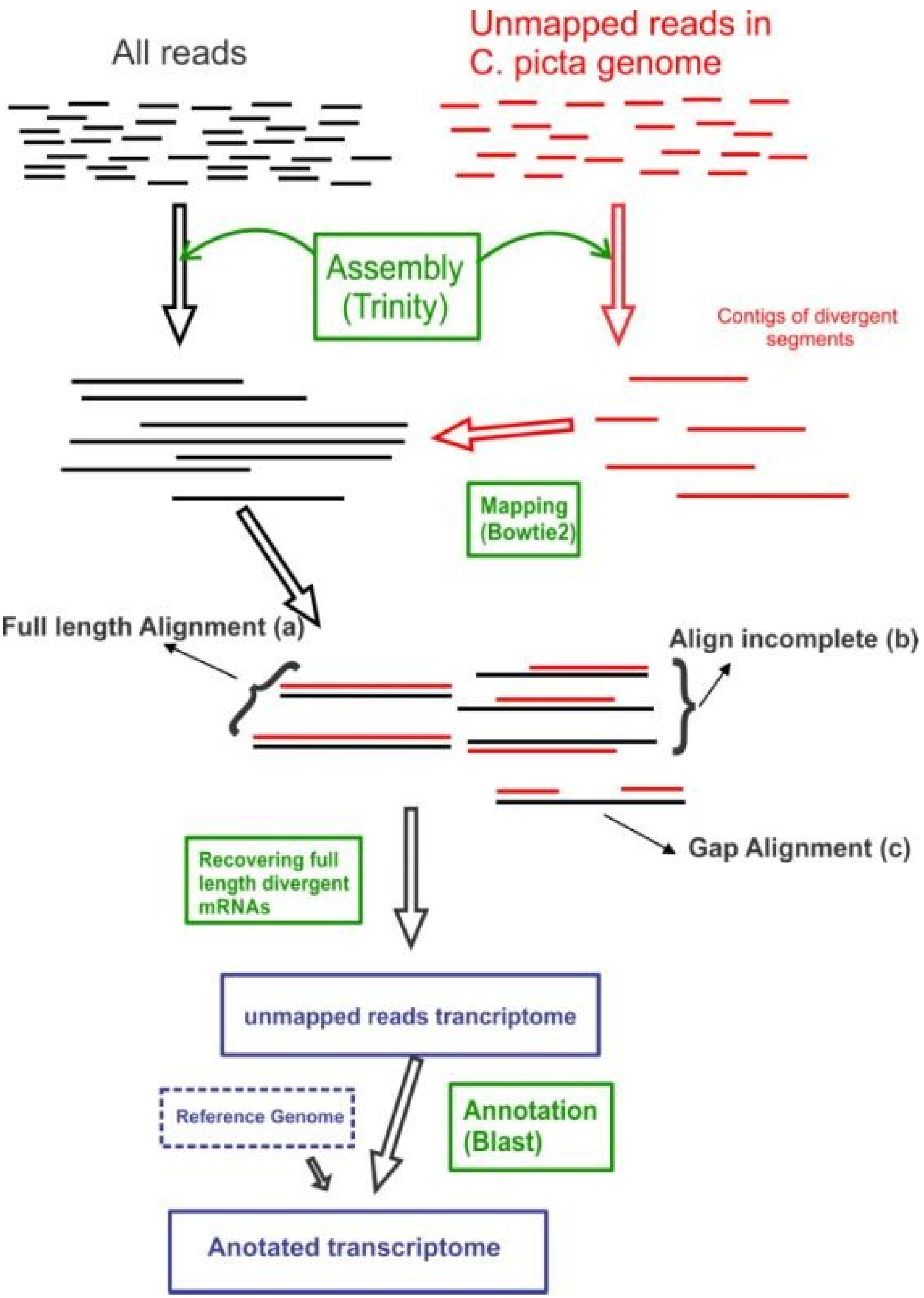

Figure SF4 Workflow of the strategy used to identify, isolate and annotate the full length transcripts corresponding to divergent (as well as species specific) genes.

Figure SF5

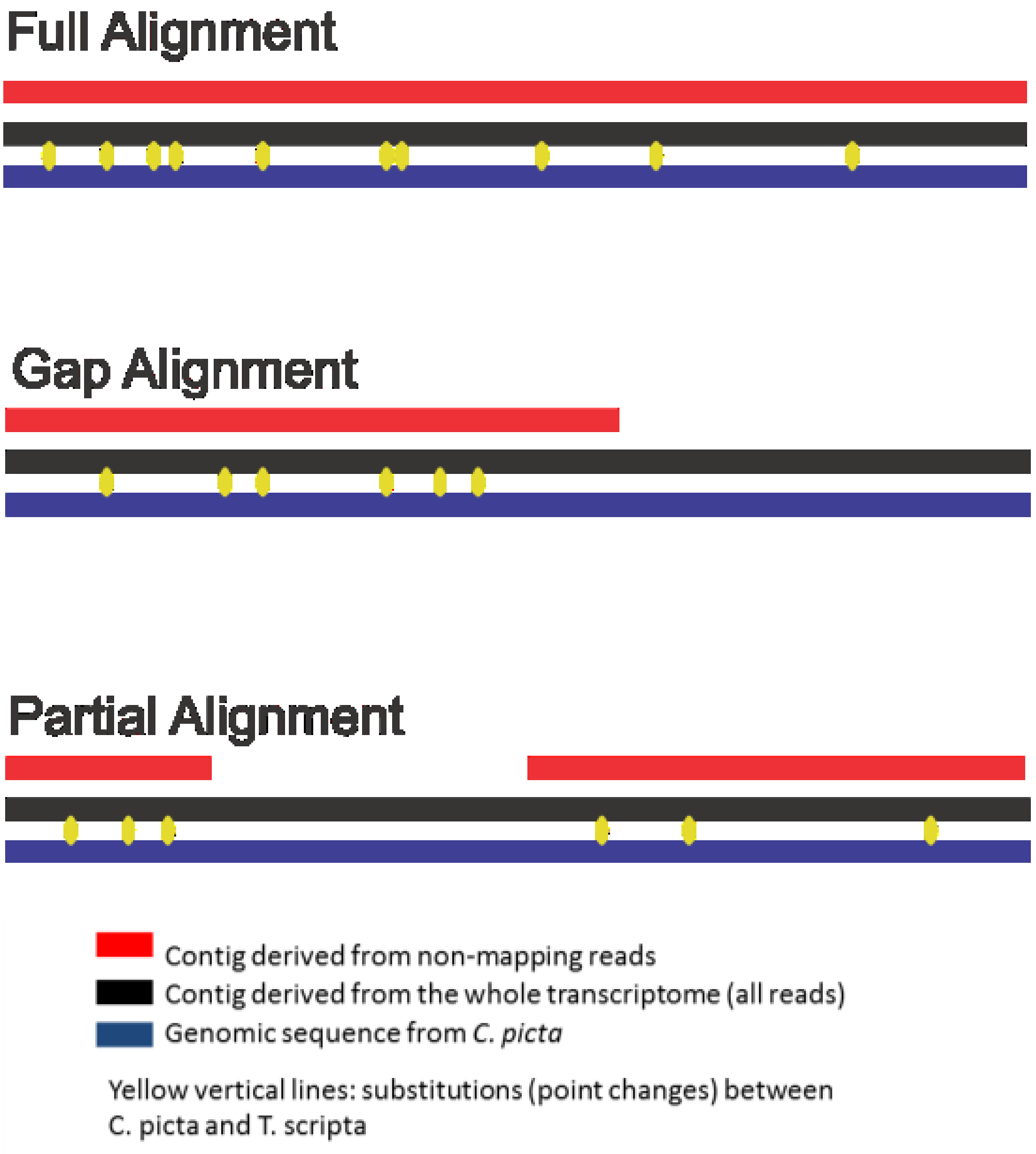

Figure SF5. Schematic representation of alignments between *T. scipta* reconstructed transcripts derived from unmmaped reads (red), reconstructed transcripts from the whole transcriptome (i.e all reads) (black line) and *C. picta* CDS homologs (blue line) The prediction of the matching patterns between RNAseq derived contigs (from T. *scripta*) and the genomic sequences from *C. picta* are indicated. The three situations correspond to those shown in figure SF6.

Figure SF6. Alignments of actual sequences illustrative examples of the situations ***a*, *b*** and ***c*** depicted in figure ***SF5***

Figure SF6.1 gene FGF19

```
Query  53   ATATTCTACATATTACTACTGGAAAAGCTAAATAATATCCTGGTTCTGGTAATGTTTCTT  112
            ||||||||||||||||||||||||||||||||||||||||||||||||||||||||||||
Sbjct  1    ATATTCTACATATTACTACTGGAAAAGCTAAATAATATCCTGGTTCTGGTAATGTTTCTT  60

Query  113  TAAAACCATATTTAAAGTCAGGAGTATTAGTTATTTCTGAAAGCTGGGGCTCTTCACTGG  172
            ||||||||||||||||||||||||||||||||||||||||||||||||||||||||||||
Sbjct  61   TAAAACCATATTTAAAGTCAGGAGTATTAGTTATTTCTGAAAGCTGGGGCTCTTCACTGG  120

Query  173  AGATATTTTAGAGGTGATTCCAAAGGGGTCCATGCTGTCAGTTTCAAGAGGTGAAGAGAA  232
            ||||||||||||||||||||||||||||||||||||||||||||||||||||||||||||
Sbjct  121  AGATATTTTAGAGGTGATTCCAAAGGGGTCCATGCTGTCAGTTTCAAGAGGTGAAGAGAA  180

Query  233  TAGATCTGATTCAAAAGTATGACTATAATCACCATATTCCACAAAATCCATTGACTCTAC  292
            ||||||||||||||||||||||||||||||||||||||||||||||||||||||||||||
Sbjct  181  TAGATCTGATTCAAAAGTATGACTATAATCACCATATTCCACAAAATCCATTGACTCTAC  240

Query  293  AGGCACGGTGTTGATCATAGGTAAGAAGTGAGACAATGGAAGAAAGTCTTTCCCTTTGAA  352
            ||||||||||||||||||||||||||||||||||||||||||||||||||||||||||||
Sbjct  241  AGGCACGGTGTTGATCATAGGTAAGAAGTGAGACAATGGAAGAAAGTCTTTCCCTTTGAA  300

Query  353  TTGTTGTCTTTGTTTGGCACTACTTAAAGACACAGAGACTCCATATTTCTTTGATTTATA  412
            ||||||||||||||||||||||||||||||||||||||||||||||||||||||||||||
Sbjct  301  TTGTTGTCTTTGTTTGGCACTACTTAAAGACACAGAGACTCCATATTTCTTTGATTTATA  360

Query  413  TACATTGTAGCCGTCTGGGCGTATCTCCTCTTCAAAGGAGCAATCTTCTGTAGAATACCT  472
            ||||||||||||||||||||||||||||||||||||||||||||||||||||||||||||
Sbjct  361  TACATTGTAGCCGTCTGGGCGTATCTCCTCTTCAAAGGAGCAATCTTCTGTAGAATACCT  420

Query  473  GAGGAGTCCATGCAGCTTCCCGTCTTCTTCCATGCAGAGGTACCGGGAACTGTGGACGCC  532
            ||||||||||||||||||||||||||||||||||||||||||||||||||||||||||||
Sbjct  421  GAGGAGTCCATGCAGCTTCCCGTCTTCTTCCATGCAGAGGTACCGGGAACTGTGGACGCC  480

Query  533  CTTGATCGCCACCGTGCGAACTGCCACCGCCCTGATTTCCAGCAGAC  579
            |||||||||||||||||||||||||||||||||||||||||||||||
Sbjct  481  CTTGATCGCCACCGTGCGAACTGCCACCGCCCTGATTTCCAGCAGAC  527
```

Figure SF6.1 Example of non-divergent alignment between a T. scripta transcriptomic contig and a *C. picta* genome sequence (Fibroblast growth factor 19, FGF19). Identities = 527/527 (100%), Gaps = 0/527 (0%)

Figure SF6.2. Gene ANGPTL2

```
Query  150    ACTGCAGCCTATTGTCCCTCCATGCCTATAGCATGAGAATGTGCATCTTAGAGTCAGCAA  209
              ||||||||||||||||||||||||||||||||||||| ||||||||||||||||||||||
Sbjct  1      ACTGCAGCCTATTGTCCCTCCATGCCTATAGCATGAGGATGTGCATCTTAGAGTCAGCAA  60

Query  210    CTGAGCTGTCtttttttCTATCAAGGAAAAACATTTAAACAATAAGAAAAACAAGATTAG  269
              ||||||||||||||||||||||||||||||||||||||||||||||||||||||||||||
Sbjct  61     CTGAGCTGTCTTTTTTTCTATCAAGGAAAAACATTTAAACAATAAGAAAAACAAGATTAG  120

Query  270    CAGAGCCTTAGGGTATTAACTTTTCTAAAGACAGTATCAAATGTATAAAACTTTTCAGTG  329
              |||||||||||||||||||||||||||| |||||||||||||||||||||||||||||||
Sbjct  121    CAGAGCCTTAGGGTATTAACTTTTCTAATGACAGTATCAAATGTATAAAACTTTTCAGTG  180

Query  330    GCAGTCTAGCTAGCTTTCACTGCAGACATACTTCTGATACAGAGATGTCACAGTTCCCAT  389
              ||||||||||||||||||||||||||||||||||||||||||||||||||||||||||||
Sbjct  181    GCAGTCTAGCTAGCTTTCACTGCAGACATACTTCTGATACAGAGATGTCACAGTTCCCAT  240

Query  390    GGATAAACTGTTCAGTTAATCTTTGATAAAAAGCACGTTGAAGACTGAAGAAAGAAGCCA  449
              ||||||||||||||||||||||||||||||||||||||| ||||||||||||||||||||
Sbjct  241    GGATAAACTGTTCAGTTAATCTTTGATAAAAAGCACGTTAAAGACTGAAGAAAGAAGCCA  300

Query  450    AAGGCAAAGGAAACACGATTTTAGAAGTTCATTTGTAGAAGAAAAAGTATCCAAAAATAA  509
              ||||||||||||||||||||||||||||||||||||||||||||||||||||||||||||
Sbjct  301    AAGGCAAAGGAAACACGATTTTAGAAGTTCATTTGTAGAAGAAAAAGTATCCAAAAATAA  360

Query  510    AGAATGATGAAGGCAGCAAGATTAACTTGTTTATTACTAATTGCAGTCATGGGAGTGACT  569
              ||||||||||||||||||||||||||||||||||||||||||||||||||||||||||||
Sbjct  361    AGAATGATGAAGGCAGCAAGATTAACTTGTTTATTACTAATTGCAGTCATGGGAGTGACT  420

Query  570    GCTAGCAAGACACAGGAATTTGAAAGCAGTGACGAGGGCACAGAGCAAGAATTCATTTAT  629
              ||||||||||||||||||||||||||||||||||||||||||||||||||||||||||||
Sbjct  421    GCTAGCAAGACACAGGAATTTGAAAGCAGTGACGAGGGCACAGAGCAAGAATTCATTTAT  480

Query  630    CTGAACAGGTATAAGCGGTCCAGCGACACACAGGACAAGTGCACTTATACCTTTATTGTA  689
              ||||||||||||||||||||||||||||||||||||||||||||||||||||||||||||
Sbjct  481    CTGAACAGGTATAAGCGGTCCAGCGACACACAGGACAAGTGCACTTATACCTTTATTGTA  540

Query  690    CCTCAGCAGAAAGTGACAGGTGCCATTTGTGTTAATTCAAAGGAGCCTGAGGTTCTTCTT  749
              ||||||||||||||||||||||||||||||||||||||||||||||||||||||||||||
Sbjct  541    CCTCAGCAGAAAGTGACAGGTGCCATTTGTGTTAATTCAAAGGAGCCTGAGGTTCTTCTT  600

Query  750    GAAAACCGGGTAAATAAACAAGAATTAGAACTGCTTAACAATGAACTTCTAAAGCAAAAG  809
              ||||||||||||||||||||||||||||| ||||||||||||||||||||||||||||||
Sbjct  601    GAAAACCGGGTAAATAAACAAGAATTAGAGCTGCTTAACAATGAACTTCTAAAGCAAAAG  660

Query  810    AGACAAATAGAAACTCTTCAGCAACTGGTGGAGGTGGATGGTGGGATTGTTAATGAGGTT  869
              ||||||||||||||||||||||||||||||||||||||||||||||||||||||||||||
Sbjct  661    AGACAAATAGAAACTCTTCAGCAACTGGTGGAGGTGGATGGTGGGATTGTTAATGAGGTT  720
```

Figure SF6.2. Gene ANGPTL2 (cont)

```
Query  870   AAACTTTTACGAAAAGAAAGTCGGAATATGAACTCTCGTGTCACACAACTTTATATGCAG  929
             ||||||||||||||||||||||||||||||||||||||||||||||||||||||||||||
Sbjct  721   AAACTTTTACGAAAAGAAAGTCGGAATATGAACTCTCGTGTCACACAACTTTATATGCAG  780

Query  930   CTGCTACATGAAATTATTCGAAAACGAGACAATGCATTAGAACTTTCCCAGCTTGAAAAT  989
             ||||||||||||||||||||||||||||||||||||||||||||||||||||||||||||
Sbjct  781   CTGCTACATGAAATTATTCGAAAACGAGACAATGCATTAGAACTTTCCCAGCTTGAAAAT  840

Query  990   AAGATATTGAACCAAACTGCTGATATGTTGCAACTTGCAAACAAATACAAGGACCTAGAG  1049
             ||||||||||||||||||||||||||||||||||||||||||||||||||||||||||||
Sbjct  841   AAGATATTGAACCAAACTGCTGATATGTTGCAACTTGCAAACAAATACAAGGACCTAGAG  900

Query  1050  TACAAATATCAACATTTGGTGACAATTGCCAATAACCAGTCTATAATTATTGCACAGCTA  1109
             ||||||||||||||||||||||||||||||||||||||||||||||||||||||||||||
Sbjct  901   TACAAATATCAACATTTGGTGACAATTGCCAATAACCAGTCTATAATTATTGCACAGCTA  960

Query  1110  GAAGAATATTGTCAAAGAATGCCATCCATTAAGCCAATACCCCAACCTCCCCAACCACCT  1169
             |||||| |||||||||||||||||||||||||||||||||||||||||||||||||||||
Sbjct  961   GAAGAACATTGTCAAAGAATGCCATCCATTAAGCCAATACCCCAACCTCCCCAACCACCT  1020

Query  1170  AACAGAATGTATCAGCCTCCTACTTACAATCGCATTATTAATCAAATAGCTACCAATGAG  1229
             ||||||||||||||||||||||||||||||||||||||||||||||||||||||||||||
Sbjct  1021  AACAGAATGTATCAGCCTCCTACTTACAATCGCATTATTAATCAAATAGCTACCAATGAG  1080

Query  1230  ATTCAAAGTGATCAGAATTTAAAGGTTCTACCACCTAATCTACCAACCATGCCACCAGTT  1289
             ||||||||||||||||||||||||||||||||||||||||||||||||||||||||||||
Sbjct  1081  ATTCAAAGTGATCAGAATTTAAAGGTTCTACCACCTAATCTACCAACCATGCCACCAGTT  1140

Query  1290  ACTAGCAGTCCAACTTCAACTGATAAACCATCTGGACCGTGGAAAGACTGTCTACAGGCA  1349
             ||||||||||||||||||||||||||||||||||||||||||||||||||||||||||||
Sbjct  1141  ACTAGCAGTCCAACTTCAACTGATAAACCATCTGGACCGTGGAAAGACTGTCTACAGGCA  1200

Query  1350  TTAGAAGATGGCCATGACACCAGTTCCATCTATCTTGTGAAACCTGAAAATACAAATCGA  1409
             ||||||||||||||||||||||||||||||||||||||||||||||||||||||||| ||
Sbjct  1201  TTAGAAGATGGCCATGACACCAGTTCCATCTATCTTGTGAAACCTGAAAATACAAATAGA  1260
```

Figure SF6.2 A: Example of alignment between *T. scripta* RNAseq contig and *C. picta* ANGPTL2 gene (Angiopoietin-related protein 2). Identity is 99%, therefore also in this case reads coming from *T. scripta* will map correctly on this *C. picta* genome.

Figure SF6.3. gene AADA

```
Query  400    AGATGGAACCACTATTGAA-ATTGGGGAGGCATTAATGAAGAGAGCCCTCCAGTATTCCT  458
              |||||||||  ||  |||| ||||||||||  || |||||||||||||||||||| ||
Sbjct  225    AGATGGAACTGCT-GTGAATATTGGGGAGGATTTGATGAAGAGAGCCCTCCAGTACTCTG  283

Query  459    CCACAAAAGGAATTCCAGAGTTCTTGTCCTGGCTAAAGGATTTACAGATGAGTCTCCACA  518
              || ||  ||||||||| ||||| ||||||||||||||||||||||||| |||||||||||
Sbjct  284    CCTCAGCAGGAATTCCTGAGTTGTTGTCCTGGCTAAAGGATTTACAGAAGAGTCTCCACA  343

Query  519    ATCCTCCCACTGCCAAGTACAGTCCTGATCAGGGCCAAATGGAGATATGTGTCACCACTG  578
              |||||||||||||||| ||||||||||||||||||||||||||||||||||||||||| |
Sbjct  344    ATCCTCCCACTGCCAACTACAGTCCTGATCAGGGCCAAATGGAGATATGTGTCACCACCG  403

Query  579    GCAGCCAGGACGGCTTGTCTAAAGTGTTTGATATGCTTGTCAATCCTGGAGATAATGTCC  638
              |||||||||| |||||||| ||||||||||| |||||| | || |||||||| ||  |||
Sbjct  404    GCAGCCAGGAAGGCTTGTCCAAAGTGTTTGAGATGCTTATTAACCCTGGAGACAACATCC  463

Query  639    TTTTAGAAGACCCCAGCTACTCAAGGACTCTGTCAGCACTGAAACCACTGGGTTGTAACA  698
              |||| || |  || | |||  |  |||||||  |||| || |||||  |||| || ||||
Sbjct  464    TTTTGGACGCACCTACCTATCCTGGGACTCTAGCAGCTCTAAAACCTTTGGGCTGCAACA  523

Query  699    TTATCAAAGTCCCTACTGACAAGCACGGTATTATTCCAGAAGCTCTGAAAGAAATTCTTT  758
              | ||||| || || | |||| |  |||||||||||||| |||| ||||||||||||||||
Sbjct  524    TAATCAATGTTCCCAGTGACCAATACGGTATTATTCCAAAAGCCCTGAAAGAAATTCTTT  583

Query  759    CCAGGTGGAGACCAGAAGACGCAAAAAAGCTGAACAACAATCTCCCCAAGTTCCTCTACA  818
              |||| ||||| |||||||| |  | |||||  ||||||| | |||| || ||||| ||||
Sbjct  584    CCAGATGGAGCCCAGAAGATGTCAGAAAGCACAACAACA-T-TCCCAAA-TTCCTGTACA  640

Query  819    CCGTTCCAAATGGTAGTAATCCATCTGGAACCTCATTAACTGCTGACTGCAAGAAGAAGA  878
              || ||||||||||| | |||||  ||||||||||||| ||| ||||| |||| ||| |||
Sbjct  641    CCATTCCAAATGGTGGCAATCCCACTGGAACCTCATTGACTACTGACCGCAAAAAGGAGA  700

Query  879    TATACCAGCTTGCAAGATGTTATGATTTCCTTATAATAGAAGATGATCCATATTATTTTC  938
              |||| ||||||||||||   ||| |||||||| |||||||||||||||| ||||||||||
Sbjct  701    TATATCAGCTTGCAAGACAGTATAATTTCCTTGTAATAGAAGATGATCCTTATTATTTTC  760

Query  939    TTCAGTTTGAAAAGACTAAGGCACCATCATTTCTGTCAATGGATGTTGATGGTCGAGTGA  998
              |||||||||| ||| ||  ||| ||| | || || |||||||| |||||||||||||| |
Sbjct  761    TTCAGTTTGATAAGCCTTGGGCTCCAACCTTCCTCTCAATGGACGTTGATGGTCGAGTTA  820

Query  999    TCAGATGTGACACTTTTTCTAAAATTATCTCTGCTGGGTTGAGATTAGGCTTTTTAACAG  1058
              ||||   |||| |||| ||||||||| ||||  ||||||||||| ||||||||||||| |
Sbjct  821    TCAGGACTGACTCTTTCTCTAAAATTCTCTCCTCTGGGTTGAGAATAGGCTTTTTAACTG  880


Query  1059   GCCCCAAACCTCTTATTGACAGAGTTATTCTACATATGCAGAGT-TCAACGAT--AG-CC  1114
              |||||||||| |||||||||||||||||||| ||||| || ||| ||||| ||   |  ||
```

```
Sbjct  881   GCCCCAAACCACTTATTGACAGAGTTATTCTGCATATACA-AGTCTCAACAATGCACACC  939

Query  1115  AGCACTTTCACACAGATCCTAATGTCAGAACTTCTTCACAAATGGGGACAAAAGGGTTTT  1174
             ||||||||||||||| |  | ||  || | ||||||||  | |||||| ||||||||||
Sbjct  940   AGCACTTTCACACAGCTTATGATTGCACAGCTTCTTCAGCAGTGGGGAGAAAAGGGTTTC  999

Query  1175  CTGGAGCATGTAGATAGGATAATGGAATTCTACAGGACCCAGAGAGATGCAATGCTTGCA  1234
             |||||||| ||||| ||| |  |||| |||||||||  |||| ||||||||||||||||
Sbjct  1000  CTGGAGCACGTAGACAGGGTTGTGGAGTTCTACAGGGTCCAGCGAGATGCAATGCTTGCT  1059

Query  1235  GCAGCAGACAAGTGGTTAAAAGGTTTGGCGGAGTGGAACAT-C-CCTACTGCTGGAATGT  1292
             |||||||||||||||||||||||| |||| || |||  ||| | ||| ||||||| ||||
Sbjct  1060  GCAGCAGACAAGTGGTTAAAAGGTCTGGCAGACTGG--CATGCTCCTGCTGCTGGCATGT  1117

Query  1293  TTTTATGGATTAAGATTAAGGGAATTTCCAATGCATACCAGATGATTATGGAAAATGCTT  1352
             || |||||||||| ||||||||| |||   |  || | ||| |||| |||||||| ||||
Sbjct  1118  TTCTATGGATTAAAATTAAGGGAGTTTTTGACACACAACAGCTGATCATGGAAAAAGCTT  1177

Query  1353  TGGAGAGAGGGGTGGCTTTGGTTCCTGGACAATCGTTTAGCATTGATAGCTCAGCTCCTA  1412
             || ||| ||  ||     ||||||||||   | | |||| |||||||||||||| |||||
Sbjct  1178  TGCAGAAAGAAGTATTACTGGTTCCTGGGGGAGCATTTAACATTGATAGCTCAGTTCCTA  1237

Query  1413  CTCCCTATGTCAGAGCTTCCTATTCTTT-TG-CTTCTCCAGACCAGATGGACCAGGCTTT  1470
              | | |||||||||||||| | |||| | || ||||||||| ||||||||| ||||||||
Sbjct  1238  GTTCTTATGTCAGAGCTTC-T-TTCTCTCTGTCTTCTCCAGCCCAGATGGATCAGGCTTT  1295

Query  1471  CCAGAAGCTGGCTGAGCTTATAAAAGAAACTGTCTGAA  1508
              |||| |||||||||||||||||||||| |||| ||||
Sbjct  1296  TCAGAGGCTGGCTGAGCTTATAAAAGAAGCTGTATGAA  1333

Identities = 803/966 (83%), Gaps = 14/966 (1%)
```

SF6.3 B Read mapping (cont.)

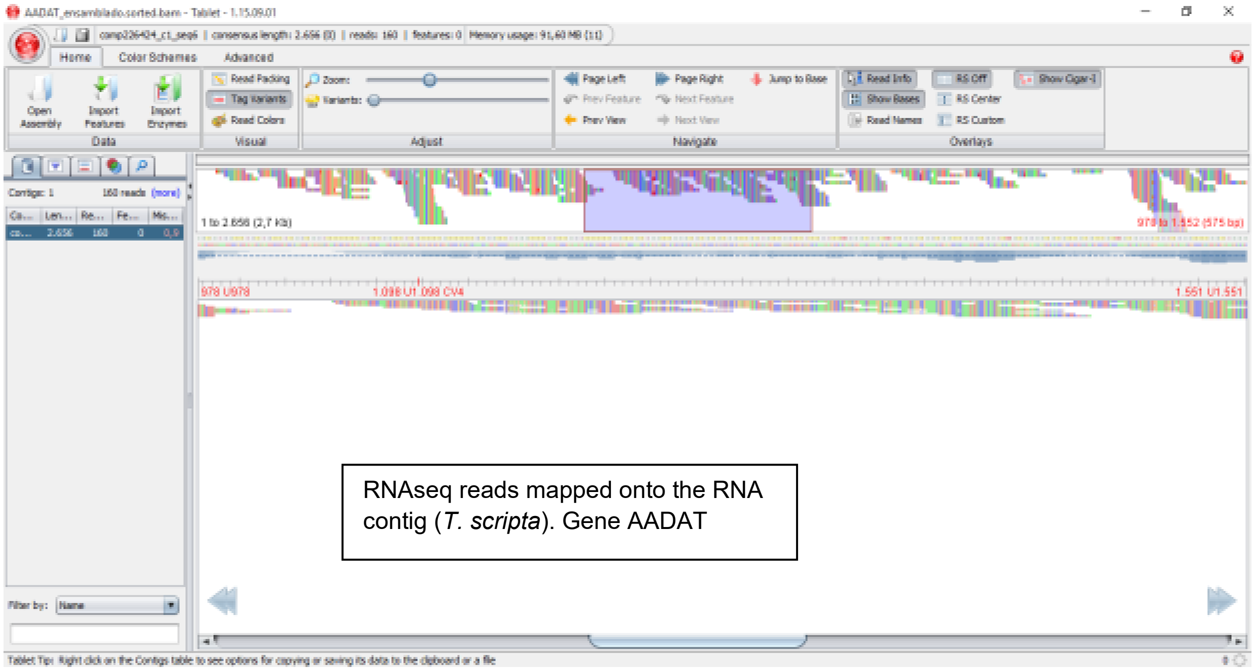

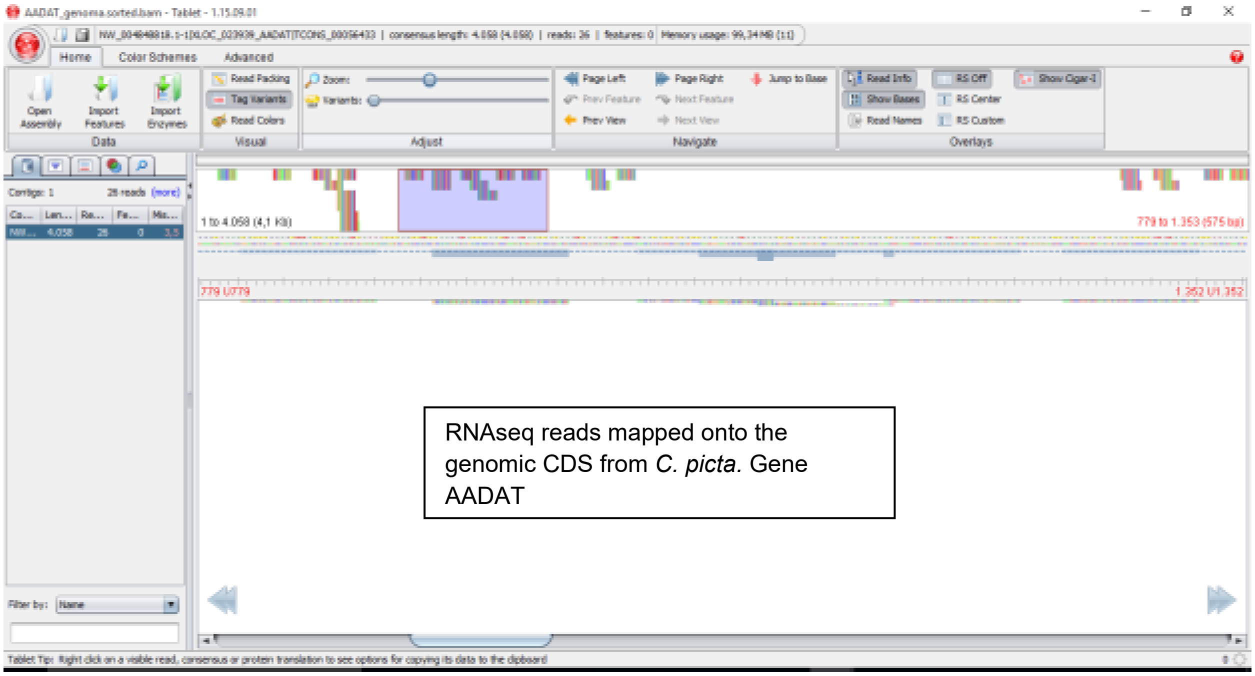

Figure SF6.3. A Alignment between *T. scripta* contig and *C. picta* gene AADAT (kynurenine/alpha-aminoadipate aminotransferase). Example of moderately divergent sequence (This example corresponds to situation **a** in the schematic depiction of figure SF5). Note that the non-matching positions between the species are scattered along the sequences, therefore a substantial proportion of RNseq reads will fail to map onto the *C. picta* genomic sequence. This is shown in panel **B** that corresponds to screenshots of mapping between reads

and *T.scripta* contig or *C. picta* gene. Note that few reads map to the genome CDS because of the divergence between species.

Figure SF6.4 A. Gene OAZ1

A

```
Query  7    TGTACGGC--AGAGTCTGGGCCGGGGGGAGGCGGCGGAGGGTTTCGGGGTTCGGACCAGA  64
            ||||||||  ||||||||||||||||||||||||||||||||||||||||||||||||||
Sbjct  1    TGTACGGCAAAGAGTCTGGGCCGGGGGGAGGCGGCGGAGGGTTTCGGGGTTCGGACCAGA  60
Query  65   GCGGCCGGATGGTGAAATCCTCCCTGCAGCGGATACTCAACAGTCACTGCTTCGCCAGAG  124
            ||||||||||||||||||||||||||||||||||||||||||||||||||||||||||||
Sbjct  61   GCGGCCGGATGGTGAAATCCTCCCTGCAGCGGATACTCAACAGTCACTGCTTCGCCAGAG  120
Query  125  AGAAAGAGGGGAATAAAAGCACCATCATCATCATGCCCGCCGTGCTGAGCCTCAGTGCCG  184
            ||||||||||||||||||||||||||||||||||||||||||||||||||||||||||||
Sbjct  121  AGAAAGAGGGGAATAAAAGCACCATCATCATCATGCCCGCCGTGCTGAGCCTCAGTGCCG  180
Query  185  GACAGAGCAGTAACTCCCAAAGTTCCAGGGTTTCTTTCAATTGCTGTAGTAACCTGGGTC  244
            |||  || ||       |  ||||||||||||||||||||||||||||||||||||||||
Sbjct  181  GAC--AG-AG-------C--AGTTCCAGGGTTTCTTTCAATTGCTGTAGTAACCTGGGTC  228
Query  245  CGGGGCCTCGGTGGTGCTCCTGATGTCCCTCACCCACCCCTGAAGATCCCAGGTGGGCGA  304
            ||||||||||||||||||||||||||||||||||||||||||||||||||||||||||||
Sbjct  229  CGGGGCCTCGGTGGTGCTCCTGATGTCCCTCACCCACCCCTGAAGATCCCAGGTGGGCGA  288
Query  305  GGGAATAGTCAGAGGGATCACAATCTTTCAGCTAATTTATTTTATTCTGATAATCGGCTG  364
            ||||||||||||||||||||||||||||||||||||||||||||||||||||||||||||
Sbjct  289  GGGAATAGTCAGAGGGATCACAATCTTTCAGCTAATTTATTTTATTCTGATAATCGGCTG  348
Query  365  AACGTAACAGAGGAACTAACATCTAATAACAGGACAAGAATTCTCAATGTCCAGTCAAGG  424
            ||||||||||||||||||||||||||||||||||||||||||||||||||||||||||||
Sbjct  349  AACGTAACAGAGGAACTAACATCTAATAACAGGACAAGAATTCTCAATGTCCAGTCAAGG  408
Query  425  CTTACAGATGCCAAACATGTTAGCTGGAGGGCAGTGCTGAACAACAACAACCTGTATATT  484
            ||||||||||||||||||||||||||||||||||||||||||||||||||||||||||||
Sbjct  409  CTTACAGATGCCAAACATGTTAGCTGGAGGGCAGTGCTGAACAACAACAACCTGTATATT  468
Query  485  GAAATTCCCAGCGGTGCTCTGCCTGAAGGGAGCAAAGACAGTTTTGCAGTTCTTCTTGAG  544
            ||||||||||||||||||||||||||||||||||||||||||||||||||||||||||||
Sbjct  469  GAAATTCCCAGCGGTGCTCTGCCTGAAGGGAGCAAAGACAGTTTTGCAGTTCTTCTTGAG  528
Query  545  TTTGCTGAAGAACAGCTTCAAGTTGATCATGTCTTCATTTGCTTCCACAAGAACAGAGAT  604
            ||||||||||||||||||||||||||||||||||||||||||||||||||||||||||||
Sbjct  529  TTTGCTGAAGAACAGCTTCAAGTTGATCATGTCTTCATTTGCTTCCACAAGAACAGAGAT  588
Query  605  GACAGAGCTGCATTGCTCCGTACTTTCAGCTTTTTGGGCTTTGAGATTGTGAGACCAGGG  664
            ||||||||||||||||||||||||||||||||||||||||||||||||||||||||||||
Sbjct  589  GACAGAGCTGCATTGCTCCGTACTTTCAGCTTTTTGGGCTTTGAGATTGTGAGACCAGGG  648
```

Figure SF6.4 B. Gene OAZ1 **Read mapping**

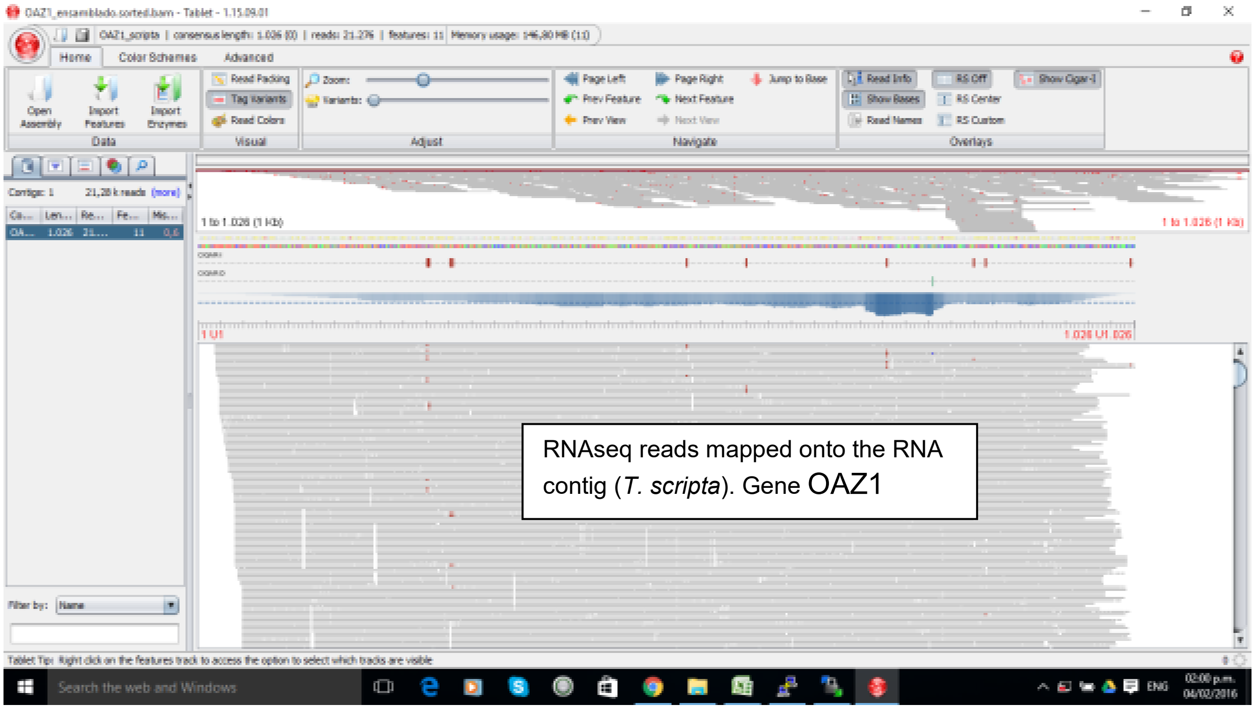

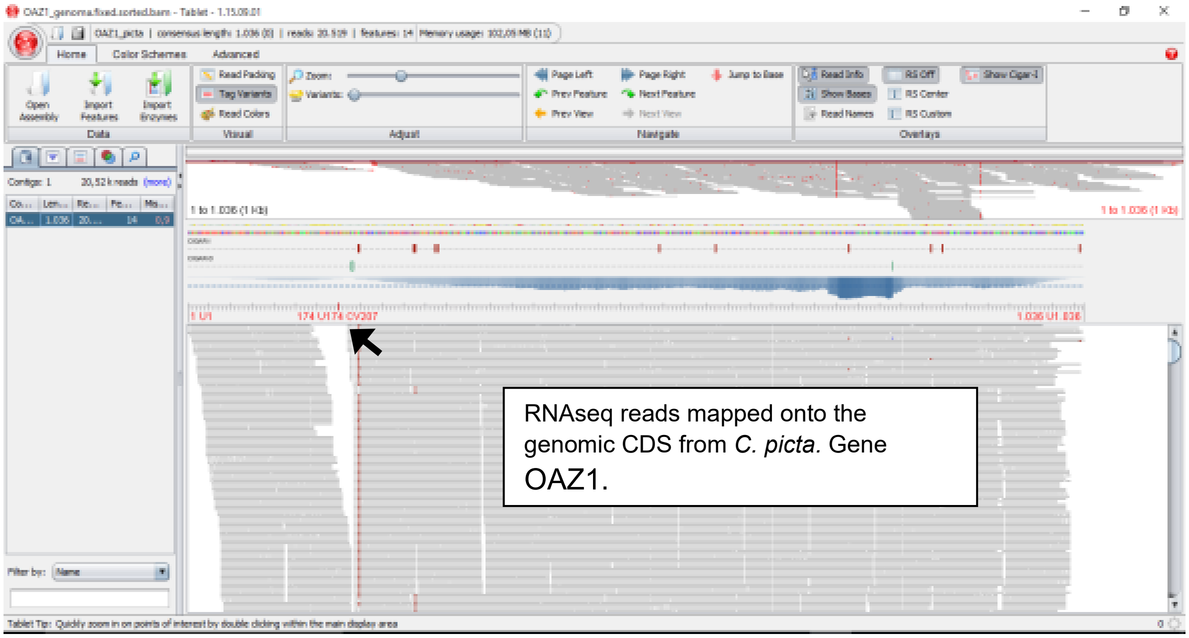

Figure SF6.4. A Alignment between *T. scripta* RNAseq contig and *C. picta* gene OAZ1. This is an example of an alignment containing a divergent segment between the two species (but he sequences are conserved in the remaining parts of the gene). Specifically the divergent region is located between bases 187 and 207 (red). RNAseq reads from *T. scripta* fail to map into this regions as shown in panel **B**, the screenshot of the mapping presented above (black arrow) thus leading to a sub-estimation of expression level.

Figure SF7

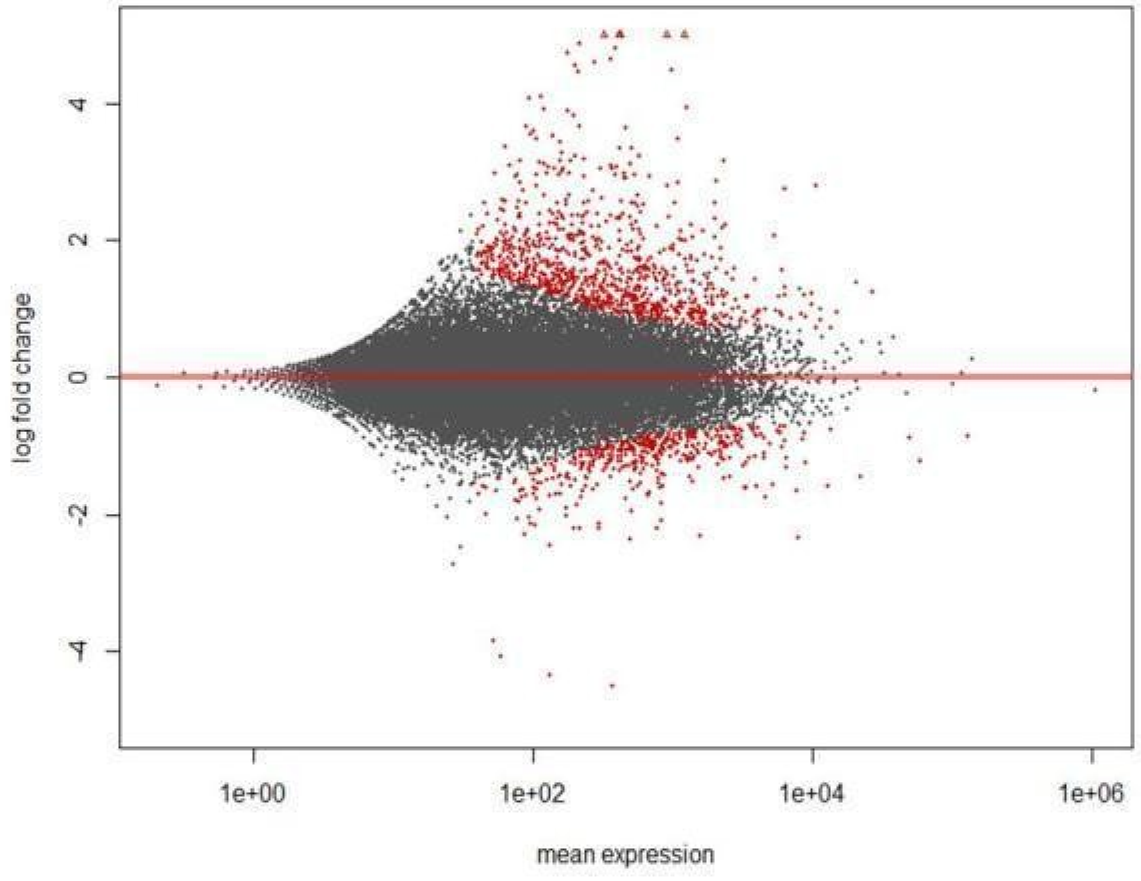

Figure SF7. MA-plot. Genes having significantly different abundance levels (FDR<0.1, FC>2) are indicated by red points.

Figure SF8

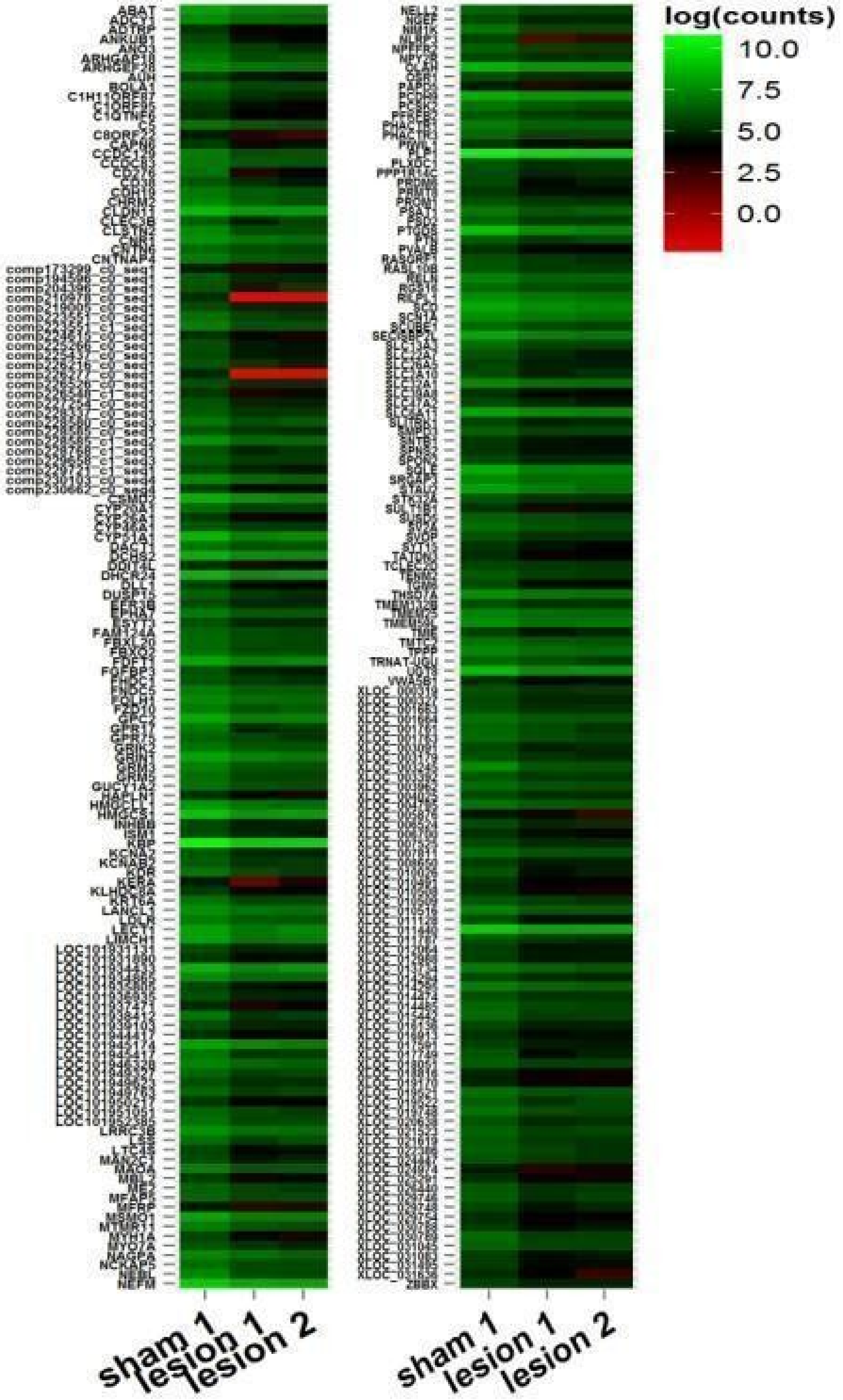

Figure SF8. Heatmap of genes that are downregulated at 4 days post lesion

Figure SF9

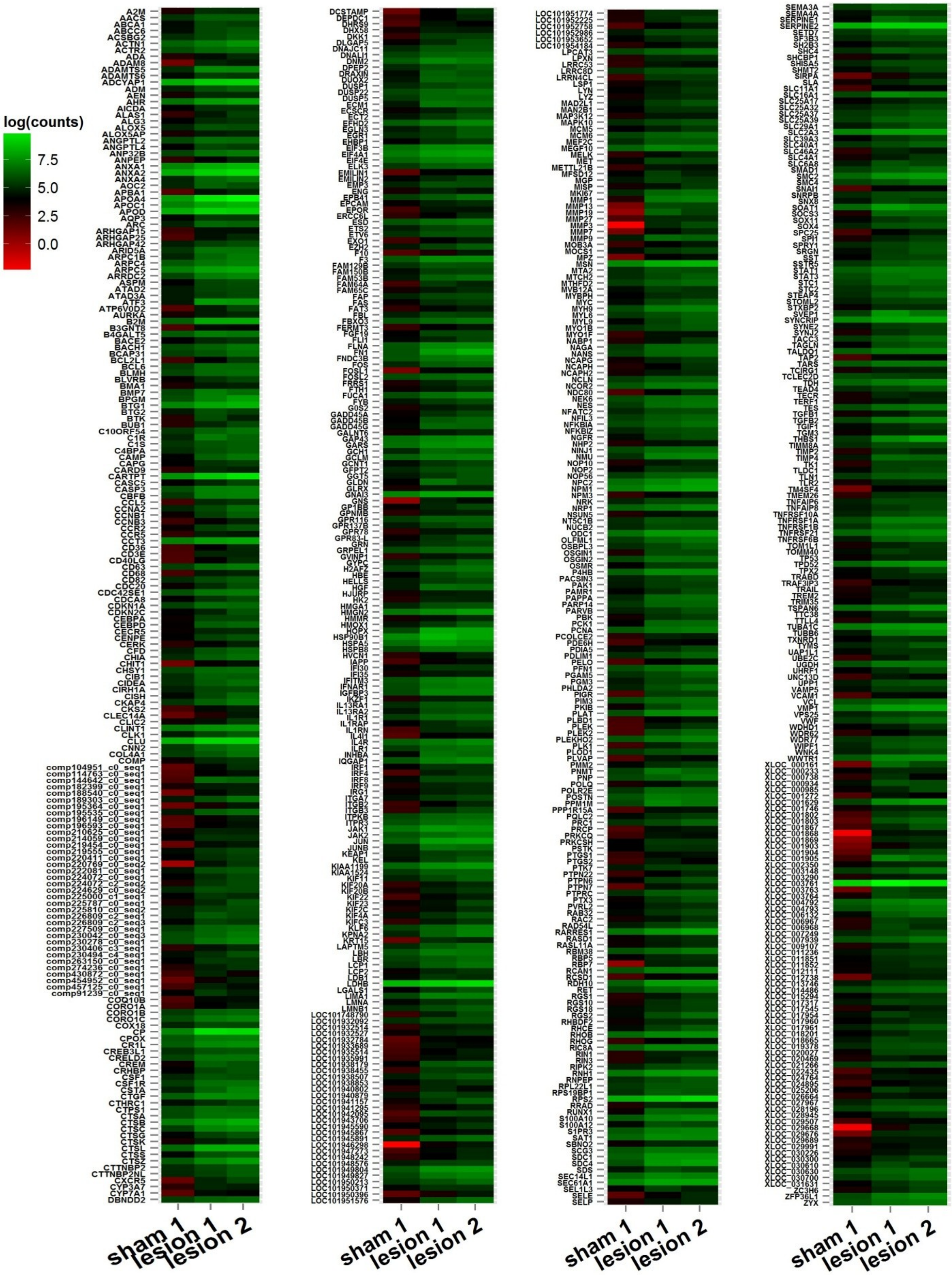

Figure SF9. Heatmap of genes that are upregulated at 4 days post lesion
